# First- and Second-Order Hypothesis Testing for Mixed Memoryless Sources

**DOI:** 10.3390/e20030174

**Published:** 2018-03-06

**Authors:** Te Sun Han, Ryo Nomura

**Affiliations:** 1 National Institute of Information and Communications Technology (NICT), Tokyo 184-8795, Japan; 2 School of Network and Information, Senshu University, Kanagawa 214-8580, Japan

**Keywords:** general source, hypothesis testing, information spectrum, mixed source, optimum exponent

## Abstract

The first- and second-order optimum achievable exponents in the simple hypothesis testing problem are investigated. The optimum achievable exponent for type II error probability, under the constraint that the type I error probability is allowed asymptotically up to ε, is called the ε-optimum exponent. In this paper, we first give the second-order ε-optimum exponent in the case where the null hypothesis and alternative hypothesis are a mixed memoryless source and a stationary memoryless source, respectively. We next generalize this setting to the case where the alternative hypothesis is also a mixed memoryless source. Secondly, we address the first-order ε-optimum exponent in this setting. In addition, an extension of our results to the more general setting such as hypothesis testing with mixed general source and a relationship with the general compound hypothesis testing problem are also discussed.

## 1. Introduction

Let $X={\left\{X^{n}\right\}}_{n=1}^{\infty }$ and $\bar{X}={\left\{{\bar{X}}^{n}\right\}}_{n=1}^{\infty }$ be two general sources (cf. Han [1]), where we use the term of general source to denote a sequence of random variables $X^{n}$ (respectively, ${\bar{X}}^{n}$) indexed by block length *n*, where each component of $X^{n}$ (respectively, ${\bar{X}}^{n}$) takes values in alphabet X and may vary depending on *n*.

We consider the hypothesis testing problem with null hypothesis X, alternative hypothesis $\bar{X}$ and acceptance region $A_{n}\subset X^{n}$. The probabilities of type I error and type II error are defined, respectively, as

(1) $$\begin{array}{c} \mu_{n}:=\mathrm{Pr}\left\{X^{n}\notin A_{n}\right\},\;\lambda_{n}:=\mathrm{Pr}\left\{{\bar{X}}^{n}\in A_{n}\right\}. \end{array}$$

We focus mainly on how to determine the ε-optimum exponent, defined as the supremum of achievable exponents *R* for the type II error probability $\lambda_{n}≃e^{-nR}$ under the constraint that the type I error probability is allowed asymptotically up to a constant ε(0≤ε<1). The classical but fundamental result in this setting is so-called Stein’s lemma [2], which gives the ε-optimum exponent in the case where both the null and alternative hypotheses are stationary memoryless sources. The lemma shows that the ε-optimum exponent is given by $D(P_{X}||P_{\bar{X}})$, the divergence between stationary memoryless sources *X* and $\bar{X}$. Chen [3] has generalized this lemma to the case where both of X and $\bar{X}$ are general sources, and established the general formula of ε-optimum exponent in terms of divergence spectra. The ε-optimum exponent derived in [3] is called in this paper the first-order ε-optimum exponent.

On the other hand, the second-order asymptotics have also been investigated in several contexts of information theory [4,5,6,7,8,9] to analyze the finer asymptotic behavior of the form $\lambda_{n}≃e^{-nR-\sqrt{n}S}$.

Strassen [4] has first introduced the notion of ε-optimum achievable exponent of the second-order in hypothesis testing problem in the case where both of X and $\bar{X}$ are stationary memoryless sources. The results in [4] have also revealed that the asymptotic normality of divergence density rate (or likelihood ratio rate) plays an important role in computing the second-order ε-optimum exponent.

In this paper, on the other hand, we investigate the hypothesis testing for mixed memoryless sources. The class of mixed sources is quite important, because all stationary sources can be regarded as mixed sources consisting of stationary ergodic sources. Therefore, the analysis for mixed sources is primitive but fundamental and thus we first focus on the case where the null hypothesis is a mixed memoryless source and the alternative hypothesis is a memoryless source. In this direction, Han [1] has first derived the single-letter formula for the first-order ε-optimum exponent in the case with mixed memoryless source X and stationary memoryless source $\bar{X}$. The first main result in this paper is to establish the single-letter second-order ε-optimum exponent in the same setting by invoking the relevant asymptotic normality. The result is a substantial generalization of that of Strassen [4]. Second, we generalize this setting to the case where both null and alternative hypotheses are mixed memoryless X, $\bar{X}$ to establish the single-letter first-order ε-optimum exponent.

It should be emphasized that our results described here are valid for mixed memoryless sources with general mixture in the sense that the mixing weight for component sources may be an arbitrary probability measure. For the case of mixed general sources with finite discrete mixture, we reveal the deep relationship with the compound hypothesis testing problem. We notice that the compound hypothesis testing problem is important from both of theoretical and practical points of view. We show that the first-order 0-optimum (respectively, exponentially *r*-optimum) exponent for the mixed general hypothesis testing coincides with that for the 0-optimum (respectively, exponentially *r*-optimum) exponent in the compound general hypothesis testing.

The present paper is organized as follows. In Section 2, we fix the problem setting and review the general formula (Theorem 1) for the first-order ε-optimum exponent. This is used to prove Theorem 5 to establish a first-order single-letter formula for hypothesis testing in the case where both the null and alternative hypotheses are mixed memoryless. Moreover, we give the general formula (Theorem 2) for the second-order ε-optimum exponent, which is used to prove Theorem 4 to establish a second-order single-letter formula for hypothesis testing in the case where the null hypothesis is mixed memoryless and the alternative hypothesis is stationary memoryless. In Section 3, we establish the single-letter second-order ε-optimum exponent in the case with mixed memoryless source X and stationary memoryless source $\bar{X}$ (cf. Theorem 4). Furthermore, in Section 4, we consider the case where both of null and alternative hypotheses are mixed memoryless sources, and derive the single-letter first-order ε-optimum exponent (cf. Theorem 5). Section 5 is devoted to an extension of mixed memoryless sources to mixed general sources. Finally, in Section 6, we define the optimum exponent for the compound general hypothesis testing problem to discuss a relevant relationship with the hypothesis testing with mixed general sources. We conclude the paper in Section 7.

## 2. General Formulas for ε-Hypothesis Testing

In this section, we first review the first-order general formula and then give the second-order general formula. Throughout in this paper, the following lemmas play the important role, where we use the notation that $P_{Z}$ indicates the probability distribution of random variable *Z*.

**Lemma** **1** ([1] (Lemma 4.1.1)). *For any t>0, define the acceptance region as*

(2) $$\begin{array}{c} A_{n}=\left\{x\in X^{n}\left|\frac{1}{n}\log\frac{P_{X^{n}}\left(x\right)}{P_{{\bar{X}}^{n}}\left(x\right)}\geq t\right.\right\}, \end{array}$$

*then, it holds that*

(3) $$\begin{array}{c} \mathrm{Pr}\left\{{\bar{X}}^{n}\in A_{n}\right\}\leq e^{-nt}. \end{array}$$

**Lemma** **2** ([1] (Lemma 4.1.2)). *For any t>0 and any $A_{n}$, it holds that*

(4) $$\begin{array}{c} \mathrm{Pr}\left\{X^{n}\notin A_{n}\right\}+e^{nt}\mathrm{Pr}\left\{{\bar{X}}^{n}\in A_{n}\right\}\geq \mathrm{Pr}\left\{\frac{1}{n}\log\frac{P_{X^{n}}\left(X^{n}\right)}{P_{{\bar{X}}^{n}}\left(X^{n}\right)}\leq t\right\}. \end{array}$$

Proofs of these lemmas are found in [1].

We define the first and second-order ε-optimum exponents as follows.

**Definition** **1.** *Rate R is said to be ε-achievable, if there exists an acceptance region $A_{n}$ such that*

(5) $$\underset{n\to \infty }{lim sup}\mu_{n}\leq \varepsilon \;and\;\underset{n\to \infty }{lim inf}\frac{1}{n}\log\frac{1}{\lambda_{n}}\geq R.$$

**Definition** **2** (First-order *ε*-optimum exponent).

(6) $$B_{\varepsilon }\left(X|\right|\bar{X}):=\sup\left\{R|R\;is\;\varepsilon -achievable\right\}.$$

The right-hand side of Equation (5) specifies the asymptotic behavior of the form $\lambda_{n}≃e^{-nR}$. Chen [3] has derived the general limiting formula for $B_{\varepsilon }\left(X|\right|\bar{X})$ as follows, which is utilized to establish Theorem 5 in Section 4.

**Theorem** **1** (Chen [3] (Theorem 1)).

(7) $$B_{\varepsilon }\left(X|\right|\bar{X})=\sup\left\{R|K\left(R\right)\leq \varepsilon \right\}\;\left(0\leq \forall \varepsilon <1\right),$$

*where*

(8) $$\begin{array}{c} K\left(R\right)=\underset{n\to \infty }{lim sup}\mathrm{Pr}\left\{\frac{1}{n}\log\frac{P_{X^{n}}\left(X^{n}\right)}{P_{{\bar{X}}^{n}}\left(X^{n}\right)}\leq R\right\}. \end{array}$$

Moreover, we consider the second-order (ε,R)-optimum exponent as follows.

**Definition** **3.** *Rate S is said to be (ε,R)-achievable, if there exists an acceptance region $A_{n}$ such that*

(9) $$\underset{n\to \infty }{lim sup}\mu_{n}\leq \varepsilon \;and\;\underset{n\to \infty }{lim inf}\frac{1}{\sqrt{n}}\log\frac{1}{\lambda_{n}e^{nR}}\geq S.$$

**Definition** **4** (Second-order (ε,R)-optimum exponent).

(10) $$B_{\varepsilon }\left(R|X|\right|\bar{X}):=\sup\left\{S|S\;is\;(\varepsilon ,R)-achievable\right\}.$$

The right-hand side of Equation (9) specifies the asymptotic behavior of the form $\lambda_{n}≃e^{-nR-\sqrt{n}S}$. The general limiting formula for $B_{\varepsilon }\left(R|X|\right|\bar{X})$ is given as follows, which is the second-order counterpart of Theorem 1, and is utilized to establish Theorem 4 in the next Section 3.2 to give a second-order single-letter formula for hypothesis testing in the case where the null hypothesis is mixed memoryless and the alternative hypothesis is stationary memoryless.

**Theorem** **2.** (11) $$B_{\varepsilon }\left(R|X|\right|\bar{X})=\sup\left\{S|K\left(R,S\right)\leq \varepsilon \right\}\;\left(0\leq \forall \varepsilon <1\right),$$

*where*

(12) $$\begin{array}{c} K\left(R,S\right)=\underset{n\to \infty }{lim sup}\mathrm{Pr}\left\{\frac{1}{n}\log\frac{P_{X^{n}}\left(X^{n}\right)}{P_{{\bar{X}}^{n}}\left(X^{n}\right)}\leq R+\frac{S}{\sqrt{n}}\right\}. \end{array}$$

**Proof.**  See Appendix A. □

## 3. Mixed Memoryless Sources

### 3.1. First-Order ε-Optimum Exponent

In the previous section, we have demonstrated the “limiting” formulas for general hypothesis testing. In this and subsequent sections, we consider special but insightful cases and compute the optimum exponents in single-letter forms.

Let Θ be an arbitrary probability space with general probability measure w(θ)(θ∈Θ). Then, the hypothesis testing problem to be considered in this section is stated as follows:

- The null hypothesis is a mixed stationary memoryless source $X={\left\{X^{n}\right\}}_{n=1}^{\infty }$, that is, for $\forall x=\left(x_{1},\cdots ,x_{n}\right)\in X^{n}$
  (13) $$P_{X^{n}}\left(x\right)=\int_{\Theta }P_{X_{\theta }^{n}}\left(x\right)dw\left(\theta \right),$$
  where $X_{\theta }^{n}$ is a stationary memoryless source for each θ∈Θ and
  (14) $$\begin{array}{c} P_{X_{\theta }^{n}}\left(x\right)=\prod_{i=1}^{n}P_{X_{\theta }}\left(x_{i}\right) \end{array}$$
  with generic random variable $X_{\theta }\;\left(\theta \in \Theta \right)$ taking values in X.
- The alternative hypothesis is a stationary memoryless source $\bar{X}={\left\{{\bar{X}}^{n}\right\}}_{n=1}^{\infty }$ with generic random variable $\bar{X}$ taking values in X, that is,
  (15) $$\begin{array}{c} P_{{\bar{X}}^{n}}\left(x\right)=\prod_{i=1}^{n}P_{\bar{X}}\left(x_{i}\right). \end{array}$$

We assume X to be a finite alphabet hereafter.

To investigate this special case, first we introduce an expurgated parameter set on the basis of types, where the type *T* of sequence $x\in X^{n}$ is the empirical distribution of x, that is, $T={\left(N\left(x|x\right)/n\right)}_{x\in X}$ with the number N(x|x) of *i* such that $x_{i}=x\;\left(i=1,2,\cdots ,n\right)$.

Let $T_{1},T_{2},\cdots ,T_{N_{n}}$ denote all possible types of sequences of length *n*. Then, it is well-known that

(16) $$N_{n}\leq {\left(n+1\right)}^{\left|X\right|}.$$

Now, for each $x\in X^{n}$, we define the set

(17) $$\Theta \left(x\right):=\left\{\theta \in \Theta \left|P_{X_{\theta }^{n}}\left(x\right)\leq e^{\sqrt[{4}]{n}}P_{X^{n}}\left(x\right)\right.\right\}.$$

Since $P_{X_{\theta }^{n}}$ is an i.i.d. source for each θ∈Θ, the set Θ(x) depends only on the type $T_{k}$ of sequence x, and therefore, we may write $\Theta (T_{k})$ instead of Θ(x). Moreover, we define the “expurgated” set

(18) $$\Theta_{n}^{*}:=\overset{N_{n}}{\underset{k=1}{⋂}}\Theta \left(T_{k}\right).$$

Then, we have the following lemma:

**Lemma** **3** (Han [1]). *Let $X={\left\{X^{n}\right\}}_{n=1}^{\infty }$ denote a mixed memoryless source defined in Equation (13), then we have*

(19) $$\int_{\Theta_{n}^{*}}dw\left(\theta \right)\geq 1-{\left(n+1\right)}^{\left|X\right|}e^{-\sqrt[{4}]{n}}.$$

Next, we introduce two basic “decomposition” lemmas as follows.

**Lemma** **4** (Upper Decomposition Lemma). Let $X={\left\{X^{n}\right\}}_{n=1}^{\infty }$ be a mixed memoryless source and $\bar{X}={\left\{{\bar{X}}^{n}\right\}}_{n=1}^{\infty }$ be an arbitrary general source. Then, for any $\theta \in \Theta_{n}^{*}$ and any real $z_{n}$ it holds that

(20) $$\begin{array}{c} \mathrm{Pr}\left\{\frac{1}{n}\log\frac{P_{X^{n}}\left(X_{\theta }^{n}\right)}{P_{{\bar{X}}^{n}}\left(X_{\theta }^{n}\right)}\leq z_{n}\right\}\leq \mathrm{Pr}\left\{\frac{1}{n}\log\frac{P_{X_{\theta }^{n}}\left(X_{\theta }^{n}\right)}{P_{{\bar{X}}^{n}}\left(X_{\theta }^{n}\right)}\leq z_{n}+\frac{1}{{\sqrt[{4}]{n}}^{3}}\right\}. \end{array}$$

**Proof.**  See Appendix B. □

**Lemma** **5** (Lower Decomposition Lemma). *Let $X={\left\{X^{n}\right\}}_{n=1}^{\infty }$ be a mixed memoryless source and $\bar{X}={\left\{{\bar{X}}^{n}\right\}}_{n=1}^{\infty }$ be an arbitrary general source. Then, for any θ∈Θ, $z_{n}$ and γ>0 it holds that*

(21) $$\begin{array}{c} \mathrm{Pr}\left\{\frac{1}{n}\log\frac{P_{X^{n}}\left(X_{\theta }^{n}\right)}{P_{{\bar{X}}^{n}}\left(X_{\theta }^{n}\right)}\leq z_{n}\right\}\geq \mathrm{Pr}\left\{\frac{1}{n}\log\frac{P_{X_{\theta }^{n}}\left(X_{\theta }^{n}\right)}{P_{{\bar{X}}^{n}}\left(X_{\theta }^{n}\right)}\leq z_{n}-\frac{\gamma }{\sqrt{n}}\right\}-e^{-\sqrt{n}\gamma }. \end{array}$$

**Proof.**  See Appendix C. □

These Lemmas 3–5 are used later in order to establish Theorems 3–5. First, Theorem 3 concerning the first-order ε-optimum exponent for mixed memoryless sources has earlier been given as follows:

**Theorem** **3** (First-order *ε*-optimum exponent: Han [1]). *For 0≤ε<1,*

(22) $$B_{\varepsilon }\left(X|\right|\bar{X})=\sup\left\{R\left|\int_{\{\theta |D(P_{X_{\theta }}\left|\right|P_{\bar{X}})<R\}}dw\left(\theta \right)\leq \varepsilon \right.\right\}$$

*where $D(P_{X}||P_{\bar{X}})$ denotes the Kullback–Leibler divergence between $P_{X}$ and $P_{\bar{X}}$.*

**Remark** **1.** *If* Θ *is a singleton, the above formula reduces to*

(23) $$B_{\varepsilon }\left(X|\right|\bar{X})=D(P_{X}\left|\right|P_{\bar{X}})\;\left(0\leq \forall \varepsilon <1\right),$$

*which is nothing but Stein’s lemma [2].*

**Remark** **2.** *$B_{\varepsilon }\left(X|\right|\bar{X})$ can be expressed also as*

(24) $$B_{\varepsilon }\left(X|\right|\bar{X})=\sup\left\{R\left|\int_{\{\theta |D(P_{X_{\theta }}\left|\right|P_{\bar{X}})\leq R\}}dw\left(\theta \right)\leq \varepsilon \right.\right\}.$$

*This can be verified as follows. Set*

(25) $$\begin{array}{cc} \beta_{\varepsilon } & :=\sup\left\{R\left|\int_{\{\theta |D(P_{X_{\theta }}\left|\right|P_{\bar{X}})<R\}}dw\left(\theta \right)\leq \varepsilon \right.\right\}, \end{array}$$

(26) $$\begin{array}{cc} {\tilde{\beta }}_{\varepsilon } & :=\sup\left\{R\left|\int_{\{\theta |D(P_{X_{\theta }}\left|\right|P_{\bar{X}})\leq R\}}dw\left(\theta \right)\leq \varepsilon \right.\right\}. \end{array}$$

*Then, clearly ${\tilde{\beta }}_{\varepsilon }\leq \beta_{\varepsilon }$. Here, we assume that ${\tilde{\beta }}_{\varepsilon }<\beta_{\varepsilon }$ to show a contradiction. From the assumption, there exists a constant γ>0 satisfying ${\tilde{\beta }}_{\varepsilon }+2\gamma <\beta_{\varepsilon }$. On the other hand, from the definition of $\beta_{\varepsilon }$, for any η>0*

(27) $$\begin{array}{cc} \varepsilon & \geq \int_{\{\theta |D(P_{X_{\theta }}\left|\right|P_{\bar{X}})<\beta_{\varepsilon }-\eta \}}dw\left(\theta \right) \end{array}$$

*holds. Thus, setting η<γ leads to*

(28) $$\begin{array}{cc} \varepsilon & \geq \int_{\{\theta |D(P_{X_{\theta }}\left|\right|P_{\bar{X}})<\beta_{\varepsilon }-\eta \}}dw\left(\theta \right)\;\\ & \geq \int_{\{\theta |D(P_{X_{\theta }}\left|\right|P_{\bar{X}})<\beta_{\varepsilon }-\gamma \}}dw\left(\theta \right)\;\\ & \geq \int_{\{\theta |D(P_{X_{\theta }}\left|\right|P_{\bar{X}})\leq {\tilde{\beta }}_{\varepsilon }+\gamma \}}dw\left(\theta \right)\;\\ & >\varepsilon , \end{array}$$

*which is a contradiction, where the last inequality is due to the definition of ${\tilde{\beta }}_{\varepsilon }$.*

### 3.2. Second-Order ε-Optimum Exponent

Next, we establish the second-order ε-optimum exponent for mixed sources, which is the first main result in this paper.

**Theorem** **4** (Second-order *ε*-optimum exponent). *For 0≤ε<1,*

(29) $$B_{\varepsilon }\left(R|X|\right|\bar{X})=\sup\left\{S\left|\int_{\{\theta |D(P_{X_{\theta }}\left|\right|P_{\bar{X}})<R\}}dw\left(\theta \right)+\int_{\{\theta |D(P_{X_{\theta }}\left|\right|P_{\bar{X}})=R\}}\Phi_{\theta }\left(S\right)dw\left(\theta \right)\leq \varepsilon \right.\right\},$$

*where*

(30) $$\Phi_{\theta }\left(S\right):=G\left(\frac{S}{\sqrt{V_{\theta }}}\right),$$

(31) $$G\left(x\right):=\frac{1}{\sqrt{2\pi }}\int_{-\infty }^{x}e^{-\frac{x^{2}}{2}}dx,$$

(32) $$V_{\theta }:=\sum_{x\in X}P_{X_{\theta }}\left(x\right){\left(\log\frac{P_{X_{\theta }}\left(x\right)}{P_{\bar{X}}\left(x\right)}-D(P_{X_{\theta }}\left|\right|P_{\bar{X}})\right)}^{2}.$$

**Proof.**  See Appendix D. □

**Remark** **3.** *If* Θ *is a singleton $\left(\Theta =\left\{\theta_{0}\right\}\right)$, Theorem 4 reduces to $B_{\varepsilon }\left(R|X|\right|\bar{X})=\sqrt{V_{\theta_{0}}}\Phi_{\theta_{0}}^{-1}\left(\varepsilon \right)$ for $R=B_{\varepsilon }\left(X|\right|\bar{X})$, which is originally due to Strassen [4].*

**Remark** **4.** *From Theorem 3 with $R=B_{\varepsilon }\left(X|\right|\bar{X})$, it is not difficult to verify that*

(33) $$\int_{\{\theta |D(P_{X_{\theta }}\left|\right|P_{\bar{X}})<R\}}dw\left(\theta \right)\leq \varepsilon$$

*and*

(34) $$\int_{\{\theta |D(P_{X_{\theta }}\left|\right|P_{\bar{X}})\leq R\}}dw\left(\theta \right)\geq \varepsilon .$$

*Here, let us consider the following canonical equation for S*

(35) $$\int_{\Theta }dw\left(\theta \right)\lim_{n\to \infty }\Phi_{\theta }(\sqrt{n}(B_{\varepsilon }\left(X|\right|\bar{X})-D(P_{X_{\theta }}\left|\right|P_{\bar{X}}))+S)=\varepsilon .$$

*In view of Equations (33) and (34), this equation always has a solution S=S(ε). It should be noted that if $\int_{\{\theta |D(P_{X_{\theta }}\left|\right|P_{\bar{X}})=B_{\varepsilon }\left(X|\right|\bar{X})\}}dw\left(\theta \right)=0$ holds, the solution is not unique and so S(ε)=+∞. By using the solution S(ε), it is not difficult to check that Theorem 4 with $R=B_{\varepsilon }\left(X|\right|\bar{X})$ can be expressed as*

(36) $$B_{\varepsilon }\left(R|X|\right|\bar{X})=S\left(\varepsilon \right).$$

The canonical equation is a useful expression for the second-order ε-optimum rate [7,10,11,12]. Equation (35) is the hypothesis testing counterpart of these results.

## 4. Mixed Memoryless Alternative Hypothesis

In this section, we consider the case where not only the null hypothesis but also the alternative hypothesis are mixed memoryless sources to establish the single-letter formula for the first-order ε-optimum exponent, by which we intend to generalize Theorem 3.

Let ${\left\{P_{{\bar{X}}_{\sigma }}\right\}}_{\sigma \in \Sigma }$ be a family of probability distributions on X, where Σ is a probability space with probability measure v(σ). We assume here that Σ is a compact space and $P_{{\bar{X}}_{\sigma }}$ is continuous as a function of σ∈Σ.

The hypothesis testing problem considered in this section is stated as follows:

- The null hypothesis is a mixed memoryless source $X={\left\{X^{n}\right\}}_{n=1}^{\infty }$ as defined by Equations (13) and (14) in Section 3.1.
- The alternative hypothesis is another mixed memoryless source $\bar{X}={\left\{{\bar{X}}^{n}\right\}}_{n=1}^{\infty }$, that is, for $\forall x\in X^{n}$
  (37) $$P_{{\bar{X}}^{n}}\left(x\right)=\int_{\Sigma }P_{{\bar{X}}_{\sigma }^{n}}\left(x\right)dv\left(\sigma \right),$$
  where
  (38) $$P_{{\bar{X}}_{\sigma }^{n}}\left(x\right)=\prod_{i=1}^{n}P_{{\bar{X}}_{\sigma }}\left(x_{i}\right).$$

Let us now consider, for each P∈P(X) (the set of probability distributions on X), the equation with respect to $\sigma^{\prime }\in \Sigma$ as follows:

(39) $$\begin{array}{c} D(P||{\bar{P}}_{\sigma^{\prime }})=v\text{-}\mathrm{ess}.\inf D(P||{\bar{P}}_{\sigma })\;\left(\mathrm{for}\;\mathrm{each}P\in P\left(X\right)\right) \end{array}$$

with $v\text{-}\mathrm{ess}.\inf f_{\sigma }:=\sup\left\{\beta |\mathrm{Pr}\left\{f_{\sigma }<\beta \right\}=0\right\}$ (the essential infimum of $f_{\sigma }$ with respect to v(σ)), where “Pr” is measured with respect to the probability measure v(σ).

Since the solution $\sigma^{\prime }$ of this equation depends on *P*, we may write as $\sigma^{\prime }=\sigma \left(P\right)\;$(σ(·):P(X)→Σ). Notice here that $D(P||{\bar{P}}_{\sigma })$ is continuous in $\left(P,{\bar{P}}_{\sigma }\right)$, and as we have assumed that Σ is compact and ${\bar{P}}_{\sigma }$ is continuous in σ, there indeed exists such a function σ(P). Now, to avoid technical subtleties, we assume here that the function σ(P) may be chosen so as to be continuous. For example, if we consider a special case such that Σ is a closed convex subset of P(X), then it is not difficult to verify that the function σ(P) is uniquely determined and continuous (or even differentiable), which follows from the strict convexity of $D(P||\bar{P})$ in $\left(P,\bar{P}\right)$. Another simple example will be the case that Σ is a countable set.

Hereafter, for simplicity, we write $P_{\theta },P_{\theta }^{n}$ (respectively, ${\bar{P}}_{\sigma },{\bar{P}}_{\sigma }^{n}$) instead of $P_{X_{\theta }},P_{X_{\theta }^{n}}$ (respectively, $P_{{\bar{X}}_{\sigma }},P_{{\bar{X}}_{\sigma }^{n}}$), then we have the second main result in this paper as

**Theorem** **5** (First order *ε*-optimum exponent). *For 0≤ε<1,*

(40) $$B_{\varepsilon }\left(X|\right|\bar{X})=\sup\left\{R\left|\int_{\{\theta |D(P_{\theta }\left|\right|{\bar{P}}_{\sigma (P_{\theta })})<R\}}dw\left(\theta \right)\leq \varepsilon \right.\right\}.$$

**Remark** **5.** *In the case that* Σ *is a singleton, the above theorem coincides with Theorem 3. Therefore, this theorem is a direct generalization of Theorem 3. This means also that both* Θ *and* Σ *are singletons, the theorem coincides with Stein’s lemma (see Remark 1).*

**Remark** **6.** *Remark 2 is also valid in this theorem. That is, $B_{\varepsilon }\left(X|\right|\bar{X})$ can be expressed also as*

(41) $$B_{\varepsilon }\left(X|\right|\bar{X})=\sup\left\{R\left|\int_{\{\theta |D(P_{\theta }\left|\right|{\bar{P}}_{\sigma (P_{\theta })})\leq R\}}dw\left(\theta \right)\leq \varepsilon \right.\right\}.$$

**Proof** **of** **Theorem** **5.** To show the theorem, let $T_{\theta ,\nu }^{n}\subseteq X^{n}$ be the set of ν-typical sequence with respect to $P_{X_{\theta }}$, that is, let $T_{\theta ,\nu }^{n}$ be the set of all $x=\left(x_{1},x_{2},\cdots ,x_{n}\right)\in X^{n}$ such that

(42) $$\left|N\left(x|x\right)/n-P_{X_{\theta }}\left(x\right)\right|\leq \nu P_{X_{\theta }}\left(x\right)\;\left(\forall x\in X\right),$$

where N(x|x) is the number of *i* such that $x_{i}=x$, and ν>0 is an arbitrary constant. Then, it is well known that

(43) $$\mathrm{Pr}\left\{X_{\theta }^{n}\in T_{\theta ,\nu }^{n}\right\}\to 1\;\left(n\to \infty \right).$$

In the sequel, we use the upper and lower bounds of the probability

(44) $$\begin{array}{c} P_{{\bar{X}}^{n}}\left(x\right)=\int_{\Sigma }{\bar{P}}_{\sigma }^{n}\left(x\right)dv\left(\sigma \right) \end{array}$$

in the form

(45) $$\begin{array}{cc} \frac{1}{n}\log\frac{1}{P_{{\bar{X}}^{n}}\left(x\right)} & \geq \frac{1}{n}\log\frac{1}{{\bar{P}}_{\sigma (P_{\theta })}^{n}\left(x\right)}-\delta_{\theta }\left(\nu \right), \end{array}$$

(46) $$\begin{array}{cc} \frac{1}{n}\log\frac{1}{P_{{\bar{X}}^{n}}\left(x\right)} & \leq \frac{1}{n}\log\frac{1}{{\bar{P}}_{\sigma (P_{\theta })}^{n}\left(x\right)}+\frac{1}{n}\log\frac{1}{c_{\tau_{m}}\left(P_{\theta }\right)}+\left(\tau -\delta_{\theta }\left(\nu \right)\right), \end{array}$$

for each $x\in T_{\theta ,\nu }^{n}$, where $\delta_{\theta }\left(\nu \right)$ satisfies $\delta_{\theta }\left(\nu \right)\to 0$ as ν→0, and τ>0 and $c_{\tau_{m}}\left(P_{\theta }\right)>0$ are some constants independent of *n*. Proofs of Equations (45) and (46) appear in Appendix E.

We then prove the theorem by using Equations (45) and (46) as follows. In view of Theorem 1 and Remark 6, it suffices to show two inequalities:

(47) $$\underset{n\to \infty }{lim sup}\mathrm{Pr}\left\{\frac{1}{n}\log\frac{P_{X^{n}}\left(X^{n}\right)}{P_{{\bar{X}}^{n}}\left(X^{n}\right)}\leq R\right\}\leq \int_{\{\theta |D(P_{\theta }\left|\right|{\bar{P}}_{\sigma (P_{\theta })})\leq R\}}dw\left(\theta \right),$$

(48) $$\underset{n\to \infty }{lim sup}\mathrm{Pr}\left\{\frac{1}{n}\log\frac{P_{X^{n}}\left(X^{n}\right)}{P_{{\bar{X}}^{n}}\left(X^{n}\right)}\leq R\right\}\geq \int_{\{\theta |D(P_{\theta }\left|\right|{\bar{P}}_{\sigma (P_{\theta })})<R\}}dw\left(\theta \right).$$

- *Proof of Equation (47)*:

Similar to the derivation of Equation (A23) with Lemma 4, we have

(49) $$\begin{array}{c} \underset{n\to \infty }{lim sup}\mathrm{Pr}\left\{\frac{1}{n}\log\frac{P_{X^{n}}\left(X^{n}\right)}{P_{{\bar{X}}^{n}}\left(X^{n}\right)}\leq R\right\}\\ =\underset{n\to \infty }{lim sup}\int_{\Theta }dw\left(\theta \right)\mathrm{Pr}\left\{\frac{1}{n}\log\frac{P_{X^{n}}\left(X_{\theta }^{n}\right)}{P_{{\bar{X}}^{n}}\left(X_{\theta }^{n}\right)}\leq R\right\}\\ \leq \int_{\Theta }dw\left(\theta \right)\underset{n\to \infty }{lim sup}\mathrm{Pr}\left\{\frac{1}{n}\log\frac{P_{X_{\theta }^{n}}\left(X_{\theta }^{n}\right)}{P_{{\bar{X}}^{n}}\left(X_{\theta }^{n}\right)}\leq R+\frac{1}{{\sqrt[{4}]{n}}^{3}}\right\}. \end{array}$$

From the definition of the ν-typical set and Equation (45), we also have

(50) $$\begin{array}{c} \underset{n\to \infty }{lim sup}\mathrm{Pr}\left\{\frac{1}{n}\log\frac{P_{X_{\theta }^{n}}\left(X_{\theta }^{n}\right)}{P_{{\bar{X}}^{n}}\left(X_{\theta }^{n}\right)}\leq R+\frac{1}{{\sqrt[{4}]{n}}^{3}}\right\}\\ \leq \underset{n\to \infty }{lim sup}\mathrm{Pr}\left\{\frac{1}{n}\log\frac{P_{X_{\theta }^{n}}\left(X_{\theta }^{n}\right)}{P_{{\bar{X}}^{n}}\left(X_{\theta }^{n}\right)}\leq R+\frac{1}{{\sqrt[{4}]{n}}^{3}},\;X_{\theta }^{n}\in T_{\theta ,\nu }^{n}\right\}+\underset{n\to \infty }{lim sup}\mathrm{Pr}\left\{X_{\theta }^{n}\notin T_{\theta ,\nu }^{n}\right\}\\ \leq \underset{n\to \infty }{lim sup}\mathrm{Pr}\left\{\frac{1}{n}\log\frac{P_{X_{\theta }^{n}}\left(X_{\theta }^{n}\right)}{{\bar{P}}_{\sigma (P_{\theta })}^{n}\left(X_{\theta }^{n}\right)}\leq R+\frac{1}{{\sqrt[{4}]{n}}^{3}}+\delta_{\theta }\left(\nu \right),\;X_{\theta }^{n}\in T_{\theta ,\nu }^{n}\right\}\\ \leq \underset{n\to \infty }{lim sup}\mathrm{Pr}\left\{\frac{1}{n}\log\frac{P_{X_{\theta }^{n}}\left(X_{\theta }^{n}\right)}{{\bar{P}}_{\sigma (P_{\theta })}^{n}\left(X_{\theta }^{n}\right)}\leq R+\frac{1}{{\sqrt[{4}]{n}}^{3}}+\delta_{\theta }\left(\nu \right)\right\}, \end{array}$$

for any θ∈Θ. Here, we define two sets:

(51) $$\begin{array}{cc} \Theta_{1} & :=\left\{\theta \in \Theta \left|D(P_{\theta }||{\bar{P}}_{\sigma (P_{\theta })})\leq R\right.\right\}, \end{array}$$

(52) $$\begin{array}{cc} \Theta_{2} & :=\left\{\theta \in \Theta \left|D(P_{\theta }||{\bar{P}}_{\sigma (P_{\theta })})>R\right.\right\}. \end{array}$$

Then, from the definition of $\Theta_{2}$ there exists a small constant γ>0 satisfying

(53) $$D(P_{\theta }||{\bar{P}}_{\sigma (P_{\theta })})\;\geq \;R+3\gamma$$

for $\theta \in \Theta_{2}$. Thus, it holds that

(54) $$\begin{array}{c} \underset{n\to \infty }{lim sup}\mathrm{Pr}\left\{\frac{1}{n}\log\frac{P_{X_{\theta }^{n}}\left(X_{\theta }^{n}\right)}{{\bar{P}}_{\sigma (P_{\theta })}^{n}\left(X_{\theta }^{n}\right)}\leq R+\frac{1}{{\sqrt[{4}]{n}}^{3}}+\delta_{\theta }\left(\nu \right)\right\}\\ \leq \underset{n\to \infty }{lim sup}\mathrm{Pr}\left\{\frac{1}{n}\log\frac{P_{X_{\theta }^{n}}\left(X_{\theta }^{n}\right)}{{\bar{P}}_{\sigma (P_{\theta })}^{n}\left(X_{\theta }^{n}\right)}\leq D(P_{\theta }\left|\right|{\bar{P}}_{\sigma (P_{\theta })})+\frac{1}{{\sqrt[{4}]{n}}^{3}}+\delta_{\theta }\left(\nu \right)-3\gamma \right\}\\ \leq \underset{n\to \infty }{lim sup}\mathrm{Pr}\left\{\frac{1}{n}\log\frac{P_{X_{\theta }^{n}}\left(X_{\theta }^{n}\right)}{{\bar{P}}_{\sigma (P_{\theta })}^{n}\left(X_{\theta }^{n}\right)}\leq D(P_{\theta }\left|\right|{\bar{P}}_{\sigma (P_{\theta })})-\gamma \right\}, \end{array}$$

where we have used the relation $\frac{1}{{\sqrt[{4}]{n}}^{3}}<\gamma$, and $\delta_{\theta }\left(\nu \right)<\gamma$ for sufficiently large *n* and sufficiently small ν>0.

Therefore, noting that, with $X_{\theta }^{n}=\left(X_{\theta ,1},X_{\theta ,2},\cdots ,X_{\theta ,n}\right)$,

(55) $$\frac{1}{n}\log\frac{P_{X_{\theta }^{n}}\left(X_{\theta }^{n}\right)}{{\bar{P}}_{\sigma (P_{\theta })}^{n}\left(X_{\theta }^{n}\right)}=\frac{1}{n}\sum_{i=1}^{n}\log\frac{P_{X_{\theta }}\left(X_{\theta ,i}\right)}{{\bar{P}}_{\sigma (P_{\theta })}\left(X_{\theta ,i}\right)}$$

gives the arithmetic average of *n* i.i.d. variables with expectation

(56) $$E\left[\frac{1}{n}\sum_{i=1}^{n}\log\frac{P_{X_{\theta }}\left(X_{\theta }\right)}{{\bar{P}}_{\sigma (P_{\theta })}^{n}\left(X_{\theta }\right)}\right]=D(P_{\theta }\left|\right|{\bar{P}}_{\sigma (P_{\theta })}).$$

Then, the weak law of large numbers yields that for $\forall \theta \in \Theta_{2}$,

(57) $$\underset{n\to \infty }{lim sup}\mathrm{Pr}\left\{\frac{1}{n}\log\frac{P_{X_{\theta }^{n}}\left(X_{\theta }^{n}\right)}{{\bar{P}}_{\sigma (P_{\theta })}^{n}\left(X_{\theta }^{n}\right)}\leq D(P_{\theta }\left|\right|{\bar{P}}_{\sigma (P_{\theta })})-\gamma \right\}=0.$$

Thus, from Equations (54) and (57), the right-hand side of Equation (49) is upper bounded by

(58) $$\begin{array}{c} \int_{\Theta }dw\left(\theta \right)\underset{n\to \infty }{lim sup}\mathrm{Pr}\left\{\frac{1}{n}\log\frac{P_{X_{\theta }^{n}}\left(X_{\theta }^{n}\right)}{{\bar{P}}_{\sigma (P_{\theta })}^{n}\left(X_{\theta }^{n}\right)}\leq R+\frac{1}{{\sqrt[{4}]{n}}^{3}}+\delta_{\theta }\left(\nu \right)\right\}\\ \leq \int_{\Theta_{1}}dw\left(\theta \right)\underset{n\to \infty }{lim sup}\mathrm{Pr}\left\{\frac{1}{n}\log\frac{P_{X_{\theta }^{n}}\left(X_{\theta }^{n}\right)}{{\bar{P}}_{\sigma (P_{\theta })}^{n}\left(X_{\theta }^{n}\right)}\leq D(P_{\theta }\left|\right|{\bar{P}}_{\sigma (P_{\theta })})-\gamma \right\}\\ \;+\int_{\Theta_{2}}dw\left(\theta \right)\underset{n\to \infty }{lim sup}\mathrm{Pr}\left\{\frac{1}{n}\log\frac{P_{X_{\theta }^{n}}\left(X_{\theta }^{n}\right)}{{\bar{P}}_{\sigma (P_{\theta })}^{n}\left(X_{\theta }^{n}\right)}\leq D(P_{\theta }\left|\right|{\bar{P}}_{\sigma (P_{\theta })})-\gamma \right\}\\ \leq \int_{\Theta_{1}}dw\left(\theta \right)\\ =\int_{\{\theta |D(P_{\theta }\left|\right|{\bar{P}}_{\sigma (P_{\theta })})\leq R\}}dw\left(\theta \right), \end{array}$$

which completes the proof of (47).

- *Proof of Equation (48)*:

Similar to the derivation of Equation (A32) with Lemma 5, we have

(59) $$\begin{array}{c} \underset{n\to \infty }{lim sup}\mathrm{Pr}\left\{\frac{1}{n}\log\frac{P_{X^{n}}\left(X^{n}\right)}{P_{{\bar{X}}^{n}}\left(X^{n}\right)}\leq R\right\}\\ \geq \int_{\Theta }dw\left(\theta \right)\underset{n\to \infty }{lim inf}\mathrm{Pr}\left\{\frac{1}{n}\log\frac{P_{X_{\theta }^{n}}\left(X_{\theta }^{n}\right)}{P_{{\bar{X}}^{n}}\left(X_{\theta }^{n}\right)}\leq R-\frac{\gamma }{\sqrt{n}}\right\}. \end{array}$$

From the definition of the ν-typical set and Equation (46), we also have

(60) $$\begin{array}{c} \underset{n\to \infty }{lim inf}\mathrm{Pr}\left\{\frac{1}{n}\log\frac{P_{X_{\theta }^{n}}\left(X_{\theta }^{n}\right)}{P_{{\bar{X}}^{n}}\left(X_{\theta }^{n}\right)}\leq R-\frac{\gamma }{\sqrt{n}}\right\}\\ \geq \underset{n\to \infty }{lim inf}\mathrm{Pr}\left\{\frac{1}{n}\log\frac{P_{X_{\theta }^{n}}\left(X_{\theta }^{n}\right)}{P_{{\bar{X}}^{n}}\left(X_{\theta }^{n}\right)}\leq R-\frac{\gamma }{\sqrt{n}},\;X_{\theta }^{n}\in T_{\theta ,\nu }^{n}\right\}\\ \geq \underset{n\to \infty }{lim inf}\mathrm{Pr}\left\{\frac{1}{n}\log\frac{P_{X_{\theta }^{n}}\left(X_{\theta }^{n}\right)}{{\bar{P}}_{\sigma (P_{\theta })}^{n}\left(X_{\theta }^{n}\right)}\leq R-\frac{\gamma }{\sqrt{n}}-\frac{1}{n}\log\frac{1}{c_{\tau_{m}}\left(P_{\theta }\right)}-\tau +\delta_{\theta }\left(\nu \right)\right\}\\ \;-\underset{n\to \infty }{lim sup}\mathrm{Pr}\left\{X_{\theta }^{n}\notin T_{\theta ,\nu }^{n}\right\}\\ =\underset{n\to \infty }{lim inf}\mathrm{Pr}\left\{\frac{1}{n}\log\frac{P_{X_{\theta }^{n}}\left(X_{\theta }^{n}\right)}{{\bar{P}}_{\sigma (P_{\theta })}^{n}\left(X_{\theta }^{n}\right)}\leq R-\frac{\gamma }{\sqrt{n}}-\frac{1}{n}\log\frac{1}{c_{\tau_{m}}\left(P_{\theta }\right)}-\tau +\delta_{\theta }\left(\nu \right)\right\} \end{array}$$

for any θ∈Θ.

We also partition the parameter space Θ into two sets.

(61) $$\begin{array}{cc} \Theta_{1}^{\prime } & :=\left\{\theta \in \Theta \left|D(P_{\theta }||{\bar{P}}_{\sigma (P_{\theta })})<R\right.\right\}, \end{array}$$

(62) $$\begin{array}{cc} \Theta_{2}^{\prime } & :=\left\{\theta \in \Theta \left|D(P_{\theta }||{\bar{P}}_{\sigma (P_{\theta })})\geq R\right.\right\}. \end{array}$$

Then, for $\theta \in \Theta_{1}^{\prime }$, if we set ν>0 and τ>0 sufficiently small, then there exists a constant η>0 satisfying

(63) $$\begin{array}{c} R-\frac{\gamma }{\sqrt{n}}-\frac{1}{n}\log\frac{1}{c_{\tau_{m}}\left(P_{\theta }\right)}-\tau +\delta_{\theta }\left(\nu \right)>D(P_{\theta }\left|\right|{\bar{P}}_{\sigma (P_{\theta })})+\eta \;\left(\forall n>n_{0}\right). \end{array}$$

Thus, again by invoking the weak law of large numbers, we have for $\forall \theta \in \Theta_{1}^{\prime }$

(64) $$\begin{array}{c} \underset{n\to \infty }{lim inf}\mathrm{Pr}\left\{\frac{1}{n}\log\frac{P_{X_{\theta }^{n}}\left(X_{\theta }^{n}\right)}{{\bar{P}}_{\sigma (P_{\theta })}^{n}\left(X_{\theta }^{n}\right)}\leq R-\frac{\gamma }{\sqrt{n}}-\frac{1}{n}\log\frac{1}{c_{\tau_{m}}\left(P_{\theta }\right)}-\tau +\delta_{\theta }\left(\nu \right)\right\}\\ \geq \underset{n\to \infty }{lim inf}\mathrm{Pr}\left\{\frac{1}{n}\log\frac{P_{X_{\theta }^{n}}\left(X_{\theta }^{n}\right)}{{\bar{P}}_{\sigma (P_{\theta })}^{n}\left(X_{\theta }^{n}\right)}\leq D(P_{\theta }\left|\right|{\bar{P}}_{\sigma (P_{\theta })})+\eta \right\}\\ =1. \end{array}$$

Summarizing up, we obtain

(65) $$\begin{array}{c} \underset{n\to \infty }{lim sup}\mathrm{Pr}\left\{\frac{1}{n}\log\frac{P_{X^{n}}\left(X^{n}\right)}{P_{{\bar{X}}^{n}}\left(X^{n}\right)}\leq R\right\}\\ \geq \int_{\Theta }dw\left(\theta \right)\underset{n\to \infty }{lim inf}\mathrm{Pr}\left\{\frac{1}{n}\log\frac{P_{X_{\theta }^{n}}\left(X_{\theta }^{n}\right)}{{\bar{P}}_{\sigma (P_{\theta })}^{n}\left(X_{\theta }^{n}\right)}\leq R-\frac{\gamma }{\sqrt{n}}-\frac{1}{n}\log\frac{1}{c_{\tau_{m}}\left(P_{\theta }\right)}-\tau +\delta_{\theta }\left(\nu \right)\right\}\\ \geq \int_{\Theta_{1}^{\prime }}dw\left(\theta \right)\underset{n\to \infty }{lim inf}\mathrm{Pr}\left\{\frac{1}{n}\log\frac{P_{X_{\theta }^{n}}\left(X_{\theta }^{n}\right)}{{\bar{P}}_{\sigma (P_{\theta })}^{n}\left(X_{\theta }^{n}\right)}\leq D(P_{\theta }\left|\right|{\bar{P}}_{\sigma (P_{\theta })})+\eta \right\}\\ =\int_{\Theta_{1}^{\prime }}dw\left(\theta \right)\\ =\int_{\{\theta |D(P_{\theta }\left|\right|{\bar{P}}_{\sigma (P_{\theta })})<R\}}dw\left(\theta \right). \end{array}$$

This completes the proof of Equation (48). □

**Remark** **7.** *Theorem 3 is a special case of Theorem 5 when* Σ *is a singleton.*

To illustrate a significance of Theorem 5, let us now consider the special case with ε=0. Then, by virtue of Theorem 5, we have the following simplified result:

**Corollary** **1.** *In the special case of ε=0, we have*

(66) $$\begin{array}{c} B_{0}\left(X|\right|\bar{X})=w\text{-}\mathrm{ess}.\underset{\theta \in \Theta }{\inf}\;v\text{-}\mathrm{ess}.\underset{\sigma \in \Sigma }{\inf}D(P_{\theta }\left|\right|{\bar{P}}_{\sigma }). \end{array}$$

**Proof.**  The formula (40) can be written in this case as

(67) $$B_{0}\left(X|\right|\bar{X})=\sup\left\{R\left|\int_{\{\theta |D(P_{\theta }\left|\right|{\bar{P}}_{\sigma (P_{\theta })})<R\}}dw\left(\theta \right)=0\right.\right\}.$$

Let

(68) $$R_{1}<\sup\left\{R\left|\int_{\{\theta |D(P_{\theta }\left|\right|{\bar{P}}_{\sigma (P_{\theta })})<R\}}dw\left(\theta \right)=0\right.\right\},$$

then this means that

(69) $$\begin{array}{cc} R_{1} & \leq w\text{-}\mathrm{ess}.\underset{\theta \in \Theta }{\inf}D(P_{\theta }\left|\right|{\bar{P}}_{\sigma (P_{\theta })})\\ & =w\text{-}\mathrm{ess}.\underset{\theta \in \Theta }{\inf}v\text{-}\mathrm{ess}.\underset{\sigma \in \Sigma }{\inf}D(P_{\theta }\left|\right|{\bar{P}}_{\sigma }). \end{array}$$

Contrarily, let

(70) $$R_{2}>\sup\left\{R\left|\int_{\{\theta |D(P_{\theta }\left|\right|{\bar{P}}_{\sigma (P_{\theta })})<R\}}dw\left(\theta \right)=0\right.\right\},$$

then this means that

(71) $$\begin{array}{cc} R_{2} & \geq w\text{-}\mathrm{ess}.\underset{\theta \in \Theta }{\inf}D(P_{\theta }\left|\right|{\bar{P}}_{\sigma (P_{\theta })})\\ & =w\text{-}\mathrm{ess}.\underset{\theta \in \Theta }{\inf}v\text{-}\mathrm{ess}.\underset{\sigma \in \Sigma }{\inf}D(P_{\theta }\left|\right|{\bar{P}}_{\sigma }). \end{array}$$

As a consequence, (66) follows from (67), (69) and (71). □

**Remark** **8.** One may wonder if it might be possible to deal with the second-order ε-optimum problem too using the arguments as developed in the above for the first-order ε-optimum problem with mixed memoryless sources X and $\bar{X}$. To do so, however, it seems that we need some novel techniques, which remain to be studied.

## 5. Hypothesis Testing with Mixed General Sources

We have so far investigated the ε-hypothesis testing for mixed memoryless sources. In this section, we deal with more general settings such as hypothesis testings with mixed general sources, which inherits the crux of that for mixed memoryless sources (cf. Theorem 5). This leads us to a primitive but insightful “general” observation.

To do so, we consider the case where both of null hypothesis X and alternative hypothesis $\bar{X}$ are finite mixtures of general sources as follows:

- The null hypothesis is a mixed general source $X={\left\{X^{n}\right\}}_{n=1}^{\infty }$ consisting of *K* general (not necessarily memoryless) sources $X_{i}={\left\{X_{i}^{n}\right\}}_{n=1}^{\infty }\;\left(i=1,2,\cdots ,K\right)$, that is, $\forall x\in X^{n}$,
  (72) $$P_{X^{n}}\left(x\right)=\sum_{i=1}^{K}\alpha_{i}P_{X_{i}^{n}}\left(x\right),$$
  where $\alpha_{i}>0\;\left(i=1,2,\cdots ,K\right)$ and $\sum_{i=1}^{K}\alpha_{i}=1.$
- The alternative hypothesis is another mixed general source $\bar{X}={\left\{{\bar{X}}^{n}\right\}}_{n=1}^{\infty }$ consisting of *L* general (not necessarily memoryless) sources ${\bar{X}}_{j}={\left\{{\bar{X}}_{j}^{n}\right\}}_{n=1}^{\infty }\;\left(j=1,\cdots ,L\right)$, that is, $\forall x\in X^{n}$,
  (73) $$P_{{\bar{X}}^{n}}\left(x\right)=\sum_{j=1}^{L}\beta_{j}P_{{\bar{X}}_{j}^{n}}\left(x\right),$$
  where $\beta_{j}>0\;\left(j=1,2,\cdots ,L\right)$ and $\sum_{j=1}^{L}\beta_{j}=1.$

In this general setting, it is hard to derive a compact formula for the first-order ε-optimum exponent (with 0<ε<1). Instead, we can obtain the following theorem in the special case of ε=0.

**Theorem** **6.** (74) $$\begin{array}{c} B_{0}\left(X|\right|\bar{X})=\min_{1\leq i\leq K,1\leq j\leq L}B_{0}(X_{i}\left|\right|{\bar{X}}_{j}). \end{array}$$

*In particular, if $X_{i}$ and ${\bar{X}}_{j}$ are all stationary memoryless sources specified by $X_{i}\;\left(i=1,2,\cdots ,K\right)$ and ${\bar{X}}_{j}\;\left(j=1,2,\cdots ,L\right)$, respectively, then*

(75) $$B_{0}\left(X|\right|\bar{X})=\min_{1\leq i\leq K,1\leq j\leq L}D(P_{X_{i}}\left|\right|P_{{\bar{X}}_{j}}),$$

*which is a special case of Corollary 1.*

**Proof.**  See Appendix F. □

Furthermore, we can also consider the following exponentially *r*-optimum exponent in hypothesis testing with two mixed general sources X and $\bar{X}$ as above.

**Definition** **5.** *Let r>0 be any fixed constant. Rate R is said to be exponentially r-achievable if there exists an acceptance region $A_{n}$ such that*

(76) $$\underset{n\to \infty }{lim inf}\frac{1}{n}\log\frac{1}{\mu_{n}}\geq r,\;\underset{n\to \infty }{lim inf}\frac{1}{n}\log\frac{1}{\lambda_{n}}\geq R.$$

**Definition** **6** (First-order exponentially *r*-optimum exponent).

(77) $$B_{e}\left(r|X|\right|\bar{X}):=\sup\left\{R|R\;is\;exponentially\;r-achievable\right\}.$$

Then, it is not difficult to verify that a result analogous to Theorem 6 holds, which is a generalization of [1] (Remark 4.4.3):

**Theorem** **7.** (78) $$B_{e}\left(r|X|\right|\bar{X})=\min_{1\leq i\leq K,1\leq j\leq L}B_{e}\left(r\right|X_{i}\left|\right|{\bar{X}}_{j}).$$

*In particular, if the null and alternative hypotheses consist of stationary memoryless sources $X_{i}\;\left(i=1,2,\cdots ,K\right)$ and ${\bar{X}}_{j}\;\left(j=1,2,\cdots ,L\right)$, respectively, then*

(79) $$B_{e}\left(r|X|\right|\bar{X})=\min_{1\leq i\leq K,1\leq j\leq L}\underset{P:D(P||P_{X_{i}})<r}{\inf}D(P||P_{{\bar{X}}_{j}}),$$

*by virtue of Hoeffding’s theorem.*

## 6. Hypothesis Testing with Compound General Sources

In this section, let us consider the compound hypothesis testing problem with finite null hypotheses $X_{i}={\left\{X_{i}^{n}\right\}}_{n=1}^{\infty }\;\left(i=1,2,\cdots ,K\right)$ and finite alternative hypotheses ${\bar{X}}_{j}={\left\{{\bar{X}}_{j}^{n}\right\}}_{n=1}^{\infty }\;\left(j=1,2,\cdots ,L\right)$, where $X_{i}$ and ${\bar{X}}_{j}$ are general sources. As is well-known, this problem is expected to have a primitive but “general” relationship to that of mixed hypothesis at the structural level.

Specifically, the compound hypothesis testing is the problem in which a pair of general sources $\left(X_{i},{\bar{X}}_{j}\right)$ occurs as a pair (null hypothesis, alternative hypothesis), and the tester does not know which pair $\left(X_{i},{\bar{X}}_{j}\right)$ is actually working. This means that the acceptance region $A_{n}$ cannot depend on *i* and *j*. The type I error probabilities of the compound hypothesis testing are given by

(80) $$\mu_{n}^{\left(i\right)}:=\mathrm{Pr}\left\{X_{i}^{n}\notin A_{n}\right\},$$

for each general null hypothesis $X_{i}$. The type II error probabilities are also given by

(81) $$\lambda_{n}^{\left(j\right)}:=\mathrm{Pr}\left\{{\bar{X}}_{j}^{n}\in A_{n}\right\},$$

for each general alternative hypothesis ${\bar{X}}_{j}$. Then, the following achievability is of our interest.

**Definition** **7.** *Rate R is said to be 0-achievable for the compound hypothesis testing, if there exists an acceptance region $A_{n}$ such that*

(82) $$\lim_{n\to \infty }\mu_{n}^{\left(i\right)}=0\;and\;\underset{n\to \infty }{lim inf}\frac{1}{n}\log\frac{1}{\lambda_{n}^{\left(j\right)}}\geq R,$$

*for all i=1,2,⋯,K and j=1,2,⋯,L.*

**Definition** **8** (First-order 0-optimum exponent).

(83) $$B({\left\{X_{i}\right\}}_{i=1}^{K}\left|\right|{\left\{{\bar{X}}_{j}\right\}}_{j=1}^{L}):=\sup\left\{R|R\;is\;0-achievable\right\}.$$

Now, we have

**Theorem** **8.** *Assuming that $\alpha_{i}>0$ and $\beta_{j}>0$ hold for all i=1,2,⋯,K and j=1,2,⋯,L, it holds that*

(84) $$\begin{array}{c} B({\left\{X_{i}\right\}}_{i=1}^{K}\left|\right|{\left\{{\bar{X}}_{j}\right\}}_{j=1}^{L})=B({\left\{\alpha_{i},X_{i}\right\}}_{i=1}^{K}\left|\right|{\left\{\beta_{j},{\bar{X}}_{j}\right\}}_{j=1}^{L}), \end{array}$$

*where with sources Equations (72) and (73), we use here the notation*

(85) $$\begin{array}{c} B({\left\{\alpha_{i},X_{i}\right\}}_{i=1}^{K}||{\left\{\beta_{j},{\bar{X}}_{j}\right\}}_{j=1}^{L}) \end{array}$$

*to denote $B_{0}\left(X|\right|\bar{X})$ to make explicit dependence on $\alpha_{i}$, $\beta_{j}$.*

**Proof.**  See Appendix G. □

From Theorems 6 and 8, we immediately obtain the first-order 0-optimum exponent for the compound hypothesis testing as:

**Corollary** **2.** *Assuming that $\alpha_{i}>0$ and $\beta_{j}>0$ hold for all i=1,2,⋯,K and j=1,2,⋯,L, we have*

(86) $$\begin{array}{c} B({\left\{X_{i}\right\}}_{i=1}^{K}\left|\right|{\left\{{\bar{X}}_{j}\right\}}_{j=1}^{L})=\min_{1\leq i\leq K,1\leq j\leq L}B_{0}(X_{i}\left|\right|{\bar{X}}_{j}). \end{array}$$

*In particular, if $X_{i}$ and ${\bar{X}}_{j}$ are all stationary memoryless sources specified by $X_{i}$ and ${\bar{X}}_{j}$, respectively, Equation (86) reduces to*

(87) $$B({\left\{X_{i}\right\}}_{i=1}^{K}\left|\right|{\left\{{\bar{X}}_{j}\right\}}_{j=1}^{L})=\min_{1\leq i\leq K,1\leq j\leq L}D(P_{X_{i}}\left|\right|P_{{\bar{X}}_{j}}).$$

**Remark** **9.** Similar to Definition 5, we can define the exponentially r-optimum exponent also for the compound hypothesis testing problem as follows.

**Definition** **9.** *Let r>0 be any fixed constant. Rate R is said to be exponentially r-achievable for the compound hypothesis testing, if there exists an acceptance region $A_{n}$ such that*

(88) $$\begin{array}{cc} \underset{n\to \infty }{lim inf}\frac{1}{n}\log\frac{1}{\mu_{n}^{\left(i\right)}} & \geq r, \end{array}$$

(89) $$\begin{array}{cc} \underset{n\to \infty }{lim inf}\frac{1}{n}\log\frac{1}{\lambda_{n}^{\left(j\right)}} & \geq R, \end{array}$$

*for all i=1,2,⋯,K and j=1,2,⋯,L.*

**Definition** **10** (First-order exponentially *r*-optimum exponent).

(90) $$B_{e}\left(r\right|{\left\{X_{i}\right\}}_{i=1}^{K}\left|\right|{\left\{{\bar{X}}_{j}\right\}}_{j=1}^{L}):=\sup\left\{R|R\;is\;exponentially\;r-achievable\right\}.$$

Then, using an argument similar to the proof of Theorem 8, the following theorem can be shown:

**Theorem** **9.** *Let $\alpha_{i}>0$ and $\beta_{j}>0$ hold for all i=1,2,⋯,K and j=1,2,⋯,L, then it holds that*

(91) $$B_{e}\left(r\right|{\left\{X_{i}\right\}}_{i=1}^{K}\left|\right|{\left\{{\bar{X}}_{j}\right\}}_{j=1}^{L})=B_{e}\left(r\right|{\left\{\alpha_{i},X_{i}\right\}}_{i=1}^{K}\left|\right|{\left\{\beta_{j},{\bar{X}}_{j}\right\}}_{j=1}^{L}),$$

*where with sources Equations (72) and (73) we use the notation*

(92) $$B_{e}\left(r\right|{\left\{\alpha_{i},X_{i}\right\}}_{i=1}^{K}\left|\right|{\left\{\beta_{j},{\bar{X}}_{j}\right\}}_{j=1}^{L})$$

*to denote $B_{e}\left(r|X|\right|\bar{X})$ (cf. Definitions 5 and 6).*

Combining Theorems 7 and 9, we immediately obtain the following corollary:

**Corollary** **3.** *Let $\alpha_{i}>0$ and $\beta_{j}>0$ hold for all i=1,2,⋯,K and j=1,2,⋯,L, then it holds that*

(93) $$B_{e}\left(r\right|{\left\{X_{i}\right\}}_{i=1}^{K}\left|\right|{\left\{{\bar{X}}_{j}\right\}}_{j=1}^{L})=\min_{1\leq i\leq K,1\leq j\leq L}B_{e}\left(r\right|X_{i}\left|\right|{\bar{X}}_{j}).$$

*In particular, if the null and alternative hypotheses consist of stationary memoryless sources specified by $X_{i}\;\left(i=1,2,\cdots ,K\right)$ and ${\bar{X}}_{j}\;\left(j=1,2,\cdots ,L\right)$, respectively, as in Theorem 7, then*

(94) $$B_{e}\left(r\right|{\left\{X_{i}\right\}}_{i=1}^{K}\left|\right|{\left\{{\bar{X}}_{j}\right\}}_{j=1}^{L})=\min_{1\leq i\leq K,1\leq j\leq L}\underset{P:D(P||P_{X_{i}})<r}{\inf}D(P||P_{{\bar{X}}_{j}}),$$

*which corresponds to Equation (79).*

## 7. Concluding Remarks

Thus far, we have investigated the first- and second-order ε-optimum exponents in the hypothesis testing problem. First, we have studied the second-order ε-optimum problem with mixed memoryless null hypothesis and stationary memoryless alternative hypothesis. As we have shown in the analysis of the second-order ε-optimum exponent, we use, as a key property, the asymptotic normality of divergence density rate for each of the component sources. We also observe that the canonical representation, first introduced in [11], is still efficient to express the second-order ε-optimum exponent for mixed memoryless sources in the hypothesis testing problem.

The first-order ε-optimum exponent in the case with mixed memoryless null and alternative hypotheses has also been established. One may wonder whether we can apply the same approach in the derivation of the second-order ε-optimum exponent in this setting. Notice that one of our key techniques to derive the first-order ε-optimum exponent is an expansion $P_{x}$ around $P_{\theta }$. More careful evaluation of this expansion would be needed to compute the second-order ε-optimum exponent. This remains to be a future work. Our final goal is the problem of hypothesis testing in which both of null and alternative hypotheses are general stationary sources. This paper characterizes the first- and second-order performance of hypothesis testing for mixed memoryless sources as a simple but crucial step toward this goal.

Finally, the relationship between the first-order 0-optimum (respectively, exponentially *r*-optimum) exponent in the hypothesis testing with mixed general sources and the 0-optimum (respectively, exponentially *r*-optimum) exponent in the compound hypothesis testing has also been demonstrated.

## Appendix A. Proof of Theorem 2

The proof consists of two parts.

(1) Direct Part:

Set $S_{0}=\sup\left\{S|K\left(R,S\right)\leq \varepsilon \right\}$. Then, we show that $S=S_{0}-\gamma$ is (ε,R)-achievable for ∀γ>0.

Define the acceptance region $A_{n}$ as

(A1) $$\begin{array}{c} A_{n}=\left\{\frac{1}{n}\log\frac{P_{X^{n}}\left(x\right)}{P_{{\bar{X}}^{n}}\left(x\right)}>R+\frac{S}{\sqrt{n}}\right\}. \end{array}$$

Then, from Lemma 4 with $t=R+\frac{S}{\sqrt{n}}$ we have the upper bound for the type II error probability $\lambda_{n}$:

(A2) $$\begin{array}{c} \lambda_{n}=\mathrm{Pr}\left\{{\bar{X}}^{n}\in A_{n}\right\}\leq e^{-nR-\sqrt{n}S}, \end{array}$$

from which it follows that

(A3) $$\underset{n\to \infty }{lim inf}\frac{1}{\sqrt{n}}\log\frac{1}{\lambda_{n}e^{nR}}\geq S.$$

We next evaluate the type I error probability $\mu_{n}$. Noting that

(A4) $$\begin{array}{c} \mu_{n}=\mathrm{Pr}\left\{X^{n}\notin A_{n}\right\}=\mathrm{Pr}\left\{\frac{1}{n}\log\frac{P_{X^{n}}\left(X^{n}\right)}{P_{{\bar{X}}^{n}}\left(X^{n}\right)}\leq R+\frac{S}{\sqrt{n}}\right\}, \end{array}$$

we have

(A5) $$\underset{n\to \infty }{lim sup}\mu_{n}=\underset{n\to \infty }{lim sup}\mathrm{Pr}\left\{\frac{1}{n}\log\frac{P_{X^{n}}\left(X^{n}\right)}{P_{{\bar{X}}^{n}}\left(X^{n}\right)}\leq R+\frac{S}{\sqrt{n}}\right\}\leq \varepsilon ,$$

because $S=S_{0}-\gamma$ by the definition. Hence, from Equations (A3) and (A5), $S=S_{0}-\gamma$ is (ε,R)-achievable. Since γ>0 is arbitrary, the direct part has been proved.

(2) Converse Part:

Suppose that *S* is (ε,R)-achievable. Then, there exists an acceptance region $A_{n}$ such that

(A6) $$\begin{array}{c} \underset{n\to \infty }{lim sup}\mu_{n}\leq \varepsilon \;\mathrm{and}\;\underset{n\to \infty }{lim inf}\frac{1}{\sqrt{n}}\log\frac{1}{\lambda_{n}e^{nR}}\geq S. \end{array}$$

We fix this acceptance region $A_{n}$. The second inequality means that for any γ>0

(A7) $$\lambda_{n}\leq e^{-nR-\sqrt{n}\left(S-\gamma \right)}$$

holds for sufficiently large *n*. On the other hand, from Lemma 2 with $t=R+\frac{S-2\gamma }{\sqrt{n}}$ it holds that

(A8) $$\begin{array}{c} \mu_{n}+e^{nR+\sqrt{n}\left(S-2\gamma \right)}\lambda_{n}\geq \mathrm{Pr}\left\{\frac{1}{n}\log\frac{P_{X^{n}}\left(X^{n}\right)}{P_{{\bar{X}}^{n}}\left(X^{n}\right)}\leq R+\frac{S-2\gamma }{\sqrt{n}}\right\}. \end{array}$$

Substituting Equation (A7) into this inequality, we have

(A9) $$\begin{array}{c} \mu_{n}+e^{-\sqrt{n}\gamma }\geq \mathrm{Pr}\left\{\frac{1}{n}\log\frac{P_{X^{n}}\left(X^{n}\right)}{P_{{\bar{X}}^{n}}\left(X^{n}\right)}\leq R+\frac{S-2\gamma }{\sqrt{n}}\right\}, \end{array}$$

for sufficiently large *n*. Thus, we have

(A10) $$\begin{array}{c} \underset{n\to \infty }{lim sup}\mu_{n}\geq \underset{n\to \infty }{lim sup}\mathrm{Pr}\left\{\frac{1}{n}\log\frac{P_{X^{n}}\left(X^{n}\right)}{P_{{\bar{X}}^{n}}\left(X^{n}\right)}\leq R+\frac{S-2\gamma }{\sqrt{n}}\right\}. \end{array}$$

Here, from Equation (A6) we have

(A11) $$\begin{array}{c} \varepsilon \geq \underset{n\to \infty }{lim sup}\mu_{n}\geq \underset{n\to \infty }{lim sup}\mathrm{Pr}\left\{\frac{1}{n}\log\frac{P_{X^{n}}\left(X^{n}\right)}{P_{{\bar{X}}^{n}}\left(X^{n}\right)}\leq R+\frac{S-2\gamma }{\sqrt{n}}\right\}, \end{array}$$

which means that

(A12) $$\begin{array}{c} S-2\gamma \leq B_{\varepsilon }\left(R|X|\right|\bar{X}). \end{array}$$

Since γ>0 is arbitrarily, the proof of the converse part has been completed. □

## Appendix B. Proof of Lemma 4

Since $P_{X_{\theta }^{n}}\left(x\right)\leq e^{\sqrt[{4}]{n}}P_{X^{n}}\left(x\right)$ holds for $\forall \theta \in \Theta_{n}^{*}$, we have

(A13) $$\begin{array}{cc} \mathrm{Pr}\left\{\frac{1}{n}\log P_{X^{n}}\left(X_{\theta }^{n}\right)\leq z_{n}\right\} & \leq \mathrm{Pr}\left\{\frac{1}{n}\log P_{X_{\theta }^{n}}\left(X_{\theta }^{n}\right)-\frac{1}{{\sqrt[{4}]{n}}^{3}}\leq z_{n}\right\}\;\\ & =\mathrm{Pr}\left\{\frac{1}{n}\log P_{X_{\theta }^{n}}\left(X_{\theta }^{n}\right)\leq z_{n}+\frac{1}{{\sqrt[{4}]{n}}^{3}}\right\} \end{array}$$

for any $z_{n}$. By using this inequality with $z_{n}+\frac{1}{n}\log P_{{\bar{X}}^{n}}\left(X_{\theta }^{n}\right)$ instead of $z_{n}$, we have

(A14) $$\begin{array}{cc} \mathrm{Pr}\left\{\frac{1}{n}\log\frac{P_{X^{n}}\left(X_{\theta }^{n}\right)}{P_{{\bar{X}}^{n}}\left(X_{\theta }^{n}\right)}\leq z_{n}\right\} & \leq \mathrm{Pr}\left\{\frac{1}{n}\log\frac{P_{X_{\theta }^{n}}\left(X_{\theta }^{n}\right)}{P_{{\bar{X}}^{n}}\left(X_{\theta }^{n}\right)}\leq z_{n}+\frac{1}{{\sqrt[{4}]{n}}^{3}}\right\} \end{array}$$

which completes the proof. □

## Appendix C. Proof of Lemma 5

Setting γ>0, we define a set

(A15) $$\begin{array}{cc} D_{n} & =\left\{x\in X^{n}\left|\frac{1}{n}\log P_{X_{\theta }^{n}}\left(x\right)-\frac{1}{n}\log P_{X^{n}}\left(x\right)\leq -\frac{\gamma }{\sqrt{n}}\right.\right\}, \end{array}$$

for θ∈Θ. Then, it holds that

(A16) $$\begin{array}{cc} \mathrm{Pr}\left\{X_{\theta }^{n}\in D_{n}\right\} & =\sum_{x\in D_{n}}P_{X_{\theta }^{n}}\left(x\right)\;\\ & \leq \sum_{x\in D_{n}}P_{X^{n}}\left(x\right)e^{-\sqrt{n}\gamma }\;\\ & \leq e^{-\sqrt{n}\gamma }. \end{array}$$

Thus, for any real number $z_{n}$ it holds that

(A17) $$\begin{array}{c} \mathrm{Pr}\left\{\frac{1}{n}\log P_{X_{\theta }^{n}}\left(X_{\theta }^{n}\right)\leq z_{n}-\frac{\gamma }{\sqrt{n}}\right\}\;\\ =\mathrm{Pr}\left\{\frac{1}{n}\log P_{X_{\theta }^{n}}\left(X_{\theta }^{n}\right)\leq z_{n}-\frac{\gamma }{\sqrt{n}},X_{\theta }^{n}\notin D_{n}\right\}+\mathrm{Pr}\left\{\frac{1}{n}\log P_{X_{\theta }^{n}}\left(X_{\theta }^{n}\right)\leq z_{n}-\frac{\gamma }{\sqrt{n}},X_{\theta }^{n}\in D_{n}\right\}\;\\ \leq \mathrm{Pr}\left\{\frac{1}{n}\log P_{X^{n}}\left(X_{\theta }^{n}\right)\leq z_{n}\right\}+\mathrm{Pr}\left\{X_{\theta }^{n}\in D_{n}\right\}\;\\ \leq \mathrm{Pr}\left\{\frac{1}{n}\log P_{X^{n}}\left(X_{\theta }^{n}\right)\leq z_{n}\right\}+e^{-\sqrt{n}\gamma }. \end{array}$$

Hence, we obtain the inequality

(A18) $$\begin{array}{cc} \mathrm{Pr}\left\{\frac{1}{n}\log P_{X^{n}}\left(X_{\theta }^{n}\right)\leq z_{n}\right\} & \geq \mathrm{Pr}\left\{\frac{1}{n}\log P_{X_{\theta }^{n}}\left(X_{\theta }^{n}\right)\leq z_{n}-\frac{\gamma }{\sqrt{n}}\right\}-e^{-\sqrt{n}\gamma }, \end{array}$$

from which with $z_{n}+\frac{1}{n}\log P_{{\bar{X}}^{n}}\left(X_{\theta }^{n}\right)$ instead of $z_{n}$ it follows that

(A19) $$\begin{array}{cc} \mathrm{Pr}\left\{\frac{1}{n}\log\frac{P_{X^{n}}\left(X_{\theta }^{n}\right)}{P_{{\bar{X}}^{n}}\left(X_{\theta }^{n}\right)}\leq z_{n}\right\} & \geq \mathrm{Pr}\left\{\frac{1}{n}\log\frac{P_{X_{\theta }^{n}}\left(X_{\theta }^{n}\right)}{P_{{\bar{X}}^{n}}\left(X_{\theta }^{n}\right)}\leq z_{n}-\frac{\gamma }{\sqrt{n}}\right\}-e^{-\sqrt{n}\gamma } \end{array}$$

for all θ∈Θ. This completes the proof. □

## Appendix D. Proof of Theorem 4

Setting

(A20) $${\bar{B}}_{\varepsilon }\left(R,S\right):=\int_{\{\theta |D(P_{X_{\theta }}\left|\right|P_{\bar{X}})<R\}}dw\left(\theta \right)+\int_{\{\theta |D(P_{X_{\theta }}\left|\right|P_{\bar{X}})=R\}}\Phi_{\theta }\left(S\right)dw\left(\theta \right),$$

it suffices, in view of Theorem 2, to show two inequalities:

(A21) $$\begin{array}{c} {\bar{B}}_{\varepsilon }\left(R,S\right)\geq \underset{n\to \infty }{lim sup}\mathrm{Pr}\left\{\frac{1}{n}\log\frac{P_{X^{n}}\left(X^{n}\right)}{P_{{\bar{X}}^{n}}\left(X^{n}\right)}\leq R+\frac{S}{\sqrt{n}}\right\}, \end{array}$$

(A22) $$\begin{array}{c} {\bar{B}}_{\varepsilon }\left(R,S\right)\leq \underset{n\to \infty }{lim sup}\mathrm{Pr}\left\{\frac{1}{n}\log\frac{P_{X^{n}}\left(X^{n}\right)}{P_{{\bar{X}}^{n}}\left(X^{n}\right)}\leq R+\frac{S}{\sqrt{n}}\right\}. \end{array}$$

- *Proof of Equation (A21)*:

By the definitions of X and $\bar{X}$, it holds that

(A23) $$\begin{array}{c} \underset{n\to \infty }{lim sup}\mathrm{Pr}\left\{\frac{1}{n}\log\frac{P_{X^{n}}\left(X^{n}\right)}{P_{{\bar{X}}^{n}}\left(X^{n}\right)}\leq R+\frac{S}{\sqrt{n}}\right\}\\ =\underset{n\to \infty }{lim sup}\int_{\Theta }dw\left(\theta \right)\mathrm{Pr}\left\{\frac{1}{n}\log\frac{P_{X^{n}}\left(X_{\theta }^{n}\right)}{P_{{\bar{X}}^{n}}\left(X_{\theta }^{n}\right)}\leq R+\frac{S}{\sqrt{n}}\right\}\\ \leq \underset{n\to \infty }{lim sup}\int_{\Theta_{n}^{*}}dw\left(\theta \right)\mathrm{Pr}\left\{\frac{1}{n}\log\frac{P_{X^{n}}\left(X_{\theta }^{n}\right)}{P_{{\bar{X}}^{n}}\left(X_{\theta }^{n}\right)}\leq R+\frac{S}{\sqrt{n}}\right\}+\underset{n\to \infty }{lim sup}\int_{\Theta -\Theta_{n}^{*}}dw\left(\theta \right)\\ =\underset{n\to \infty }{lim sup}\int_{\Theta_{n}^{*}}dw\left(\theta \right)\mathrm{Pr}\left\{\frac{1}{n}\log\frac{P_{X^{n}}\left(X_{\theta }^{n}\right)}{P_{{\bar{X}}^{n}}\left(X_{\theta }^{n}\right)}\leq R+\frac{S}{\sqrt{n}}\right\}\\ \leq \underset{n\to \infty }{lim sup}\int_{\Theta_{n}^{*}}dw\left(\theta \right)\mathrm{Pr}\left\{\frac{1}{n}\log\frac{P_{X_{\theta }^{n}}\left(X_{\theta }^{n}\right)}{P_{{\bar{X}}^{n}}\left(X_{\theta }^{n}\right)}\leq R+\frac{S}{\sqrt{n}}+\frac{1}{{\sqrt[{4}]{n}}^{3}}\right\}\\ \leq \underset{n\to \infty }{lim sup}\int_{\Theta }dw\left(\theta \right)\mathrm{Pr}\left\{\frac{1}{n}\log\frac{P_{X_{\theta }^{n}}\left(X_{\theta }^{n}\right)}{P_{{\bar{X}}^{n}}\left(X_{\theta }^{n}\right)}\leq R+\frac{S}{\sqrt{n}}+\frac{1}{{\sqrt[{4}]{n}}^{3}}\right\}\\ \leq \int_{\Theta }dw\left(\theta \right)\underset{n\to \infty }{lim sup}\mathrm{Pr}\left\{\frac{1}{n}\log\frac{P_{X_{\theta }^{n}}\left(X_{\theta }^{n}\right)}{P_{{\bar{X}}^{n}}\left(X_{\theta }^{n}\right)}\leq R+\frac{S}{\sqrt{n}}+\frac{1}{{\sqrt[{4}]{n}}^{3}}\right\}, \end{array}$$

where the second equality and the second inequality are due to Lemmas 3 and 4, respectively, and the last inequality is from the reverse Fatou’s lemma.

Here, we define three sets:

(A24) $$\begin{array}{cc} \Theta_{0} & :=\left\{\theta \in \Theta \left|D(P_{X_{\theta }}||P_{\bar{X}})=R\right.\right\}, \end{array}$$

(A25) $$\begin{array}{cc} \Theta_{1} & :=\left\{\theta \in \Theta \left|D(P_{X_{\theta }}||P_{\bar{X}})<R\right.\right\}, \end{array}$$

(A26) $$\begin{array}{cc} \Theta_{2} & :=\left\{\theta \in \Theta \left|D(P_{X_{\theta }}||P_{\bar{X}})>R\right.\right\}. \end{array}$$

Noting that, setting $X_{\theta }^{n}=\left(X_{\theta ,1},X_{\theta ,2},\cdots ,X_{\theta ,n}\right)$,

(A27) $$\begin{array}{c} \frac{1}{n}\log\frac{P_{X_{\theta }^{n}}\left(X_{\theta }^{n}\right)}{P_{{\bar{X}}^{n}}\left(X_{\theta }^{n}\right)}=\frac{1}{n}\sum_{i=1}^{n}\log\frac{P_{X_{\theta }}\left(X_{\theta ,i}\right)}{P_{\bar{X}}\left(X_{\theta ,i}\right)} \end{array}$$

gives the arithmetic average of *n* i.i.d. variables with expectation

(A28) $$\begin{array}{c} E\left[\frac{1}{n}\sum_{i=1}^{n}\log\frac{P_{X_{\theta }}\left(X_{\theta }\right)}{P_{\bar{X}}\left(X_{\theta }\right)}\right]=D(P_{X_{\theta }}\left|\right|P_{\bar{X}}). \end{array}$$

Then, the weak law of large numbers yields that for $\forall \theta \in \Theta_{2}$

(A29) $$\underset{n\to \infty }{lim sup}\mathrm{Pr}\left\{\frac{1}{n}\log\frac{P_{X_{\theta }^{n}}\left(X_{\theta }^{n}\right)}{P_{{\bar{X}}^{n}}\left(X_{\theta }^{n}\right)}\leq R+\frac{S}{\sqrt{n}}+\frac{1}{{\sqrt[{4}]{n}}^{3}}\right\}=0.$$

Moreover, for $\forall \theta \in \Theta_{0}$, the central limit theorem leads to

(A30) $$\begin{array}{c} \underset{n\to \infty }{lim sup}\mathrm{Pr}\left\{\frac{1}{n}\log\frac{P_{X_{\theta }^{n}}\left(X_{\theta }^{n}\right)}{P_{{\bar{X}}^{n}}\left(X_{\theta }^{n}\right)}\leq R+\frac{S}{\sqrt{n}}+\frac{1}{{\sqrt[{4}]{n}}^{3}}\right\}\\ =\underset{n\to \infty }{lim sup}\mathrm{Pr}\left\{\frac{1}{\sqrt{n}}\left(\log\frac{P_{X_{\theta }^{n}}\left(X_{\theta }^{n}\right)}{P_{{\bar{X}}^{n}}\left(X_{\theta }^{n}\right)}-\sqrt{n}D(P_{X_{\theta }}\left|\right|P_{\bar{X}})\right)\leq S+\frac{1}{\sqrt[{4}]{n}}\right\}\\ =\Phi_{\theta }\left(S\right). \end{array}$$

Summarizing these equalities, we obtain

(A31) $$\begin{array}{c} \int_{\Theta }dw\left(\theta \right)\underset{n\to \infty }{lim sup}\mathrm{Pr}\left\{\frac{1}{n}\log\frac{P_{X_{\theta }^{n}}\left(X_{\theta }^{n}\right)}{P_{{\bar{X}}^{n}}\left(X_{\theta }^{n}\right)}\leq R+\frac{S}{\sqrt{n}}+\frac{1}{{\sqrt[{4}]{n}}^{3}}\right\}\\ =\int_{\Theta_{1}}dw\left(\theta \right)\underset{n\to \infty }{lim sup}\mathrm{Pr}\left\{\frac{1}{n}\log\frac{P_{X_{\theta }^{n}}\left(X_{\theta }^{n}\right)}{P_{{\bar{X}}^{n}}\left(X_{\theta }^{n}\right)}\leq R+\frac{S}{\sqrt{n}}+\frac{1}{{\sqrt[{4}]{n}}^{3}}\right\}\\ \;+\int_{\Theta_{0}}dw\left(\theta \right)\underset{n\to \infty }{lim sup}\mathrm{Pr}\left\{\frac{1}{n}\log\frac{P_{X_{\theta }^{n}}\left(X_{\theta }^{n}\right)}{P_{{\bar{X}}^{n}}\left(X_{\theta }^{n}\right)}\leq R+\frac{S}{\sqrt{n}}+\frac{1}{{\sqrt[{4}]{n}}^{3}}\right\}\\ \leq \int_{\{\theta |D(P_{X_{\theta }}\left|\right|P_{\bar{X}})<R\}}dw\left(\theta \right)+\int_{\{\theta |D(P_{X_{\theta }}\left|\right|P_{\bar{X}})=R\}}\Phi_{\theta }\left(S\right)dw\left(\theta \right). \end{array}$$

Plugging Equation (A31) into Equation (A23) yields Equation (A21).

- *Proof of Equation (A22)*:

By the definitions of X and $\bar{X}$, and Lemma 5 with $z_{n}=R+\frac{S}{\sqrt{n}}$, it holds that

(A32) $$\begin{array}{c} \underset{n\to \infty }{lim sup}\mathrm{Pr}\left\{\frac{1}{n}\log\frac{P_{X^{n}}\left(X^{n}\right)}{P_{{\bar{X}}^{n}}\left(X^{n}\right)}\leq R+\frac{S}{\sqrt{n}}\right\}\\ \geq \underset{n\to \infty }{lim inf}\int_{\Theta }dw\left(\theta \right)\mathrm{Pr}\left\{\frac{1}{n}\log\frac{P_{X^{n}}\left(X_{\theta }^{n}\right)}{P_{{\bar{X}}^{n}}\left(X_{\theta }^{n}\right)}\leq R+\frac{S}{\sqrt{n}}\right\}\\ \geq \underset{n\to \infty }{lim inf}\int_{\Theta }dw\left(\theta \right)\mathrm{Pr}\left\{\frac{1}{n}\log\frac{P_{X_{\theta }^{n}}\left(X_{\theta }^{n}\right)}{P_{{\bar{X}}^{n}}\left(X_{\theta }^{n}\right)}\leq R+\frac{S}{\sqrt{n}}-\frac{\gamma }{\sqrt{n}}\right\}\\ =\underset{n\to \infty }{lim inf}\int_{\Theta }dw\left(\theta \right)\mathrm{Pr}\left\{\frac{1}{n}\log\frac{P_{X_{\theta }^{n}}\left(X_{\theta }^{n}\right)}{P_{{\bar{X}}^{n}}\left(X_{\theta }^{n}\right)}\leq R+\frac{S-\gamma }{\sqrt{n}}\right\}\\ \geq \int_{\Theta }dw\left(\theta \right)\underset{n\to \infty }{lim inf}\mathrm{Pr}\left\{\frac{1}{n}\log\frac{P_{X_{\theta }^{n}}\left(X_{\theta }^{n}\right)}{P_{{\bar{X}}^{n}}\left(X_{\theta }^{n}\right)}\leq R+\frac{S-\gamma }{\sqrt{n}}\right\}, \end{array}$$

for any γ>0, where the last inequality is due to Fatou’s lemma. We also partition the parameter space Θ into three sets as in Equations (A24)–(A26).

Then, similarly to the derivation of Equations (A29) and (A30), we obtain

(A33) $$\underset{n\to \infty }{lim inf}\mathrm{Pr}\left\{\frac{1}{n}\log\frac{P_{X_{\theta }^{n}}\left(X_{\theta }^{n}\right)}{P_{{\bar{X}}^{n}}\left(X_{\theta }^{n}\right)}\leq R+\frac{S-\gamma }{\sqrt{n}}\right\}=\left\{\begin{array}{cc} \Phi_{\theta }\left(S-\gamma \right),\; & \theta \in \Theta_{0}\\ 1.\; & \theta \in \Theta_{1} \end{array}\right.$$

Thus, the right-hand side of Equation (A32) is rewritten as

(A34) $$\begin{array}{c} \int_{\Theta }dw\left(\theta \right)\underset{n\to \infty }{lim inf}\mathrm{Pr}\left\{\frac{1}{n}\log\frac{P_{X_{\theta }^{n}}\left(X_{\theta }^{n}\right)}{P_{{\bar{X}}^{n}}\left(X_{\theta }^{n}\right)}\leq R+\frac{S-\gamma }{\sqrt{n}}\right\}\;\\ \geq \int_{\Theta_{1}}dw\left(\theta \right)\underset{n\to \infty }{lim inf}\mathrm{Pr}\left\{\frac{1}{n}\log\frac{P_{X_{\theta }^{n}}\left(X_{\theta }^{n}\right)}{P_{{\bar{X}}^{n}}\left(X_{\theta }^{n}\right)}\leq R+\frac{S-\gamma }{\sqrt{n}}\right\}\;\\ \;+\int_{\Theta_{0}}dw\left(\theta \right)\underset{n\to \infty }{lim inf}\mathrm{Pr}\left\{\frac{1}{n}\log\frac{P_{X_{\theta }^{n}}\left(X_{\theta }^{n}\right)}{P_{{\bar{X}}^{n}}\left(X_{\theta }^{n}\right)}\leq R+\frac{S-\gamma }{\sqrt{n}}\right\}\;\\ =\int_{\{\theta |D(P_{X_{\theta }}\left|\right|P_{\bar{X}})<R\}}dw\left(\theta \right)+\int_{\{\theta |D(P_{X_{\theta }}\left|\right|P_{\bar{X}})=R\}}\Phi_{\theta }\left(S-\gamma \right)dw\left(\theta \right). \end{array}$$

Substituting Equation (A34) into Equation (A32) and noting that γ>0 is arbitrary, we obtain Equation (A22). □

## Appendix E. Proofs of Equations (45) and (46)

(1) Proof of Equation (45):

To prove Equation (45), we define a(x) as

(A35) $$a\left(x\right):=v\text{-}\mathrm{ess}.\sup{\bar{P}}_{\sigma }^{n}\left(x\right),$$

where $v\text{-}\mathrm{ess}.\sup f_{\sigma }$ denotes the essential supremum of $f_{\sigma }$ with respect to v(σ), i.e., $v\text{-}\mathrm{ess}.\sup f_{\sigma }:=\inf\left\{\alpha |\mathrm{Pr}\left\{f_{\sigma }>\alpha \right\}=0\right\}$. Thus, from the property of the essential supremum we immediately have

(A36) $$a\left(x\right)\geq P_{{\bar{X}}^{n}}\left(x\right),$$

for ∀n=1,2,⋯.

Let $P_{x}$ denote the type of $x\in T_{\theta ,\nu }^{n}$. Then, noting that

(A37) $$\begin{array}{cc} {\bar{P}}_{\sigma }^{n}\left(x\right) & =\prod_{x\in X}{\bar{P}}_{\sigma }{\left(x\right)}^{N(x|x)}\;\\ & =\exp\left[\sum_{x\in X}N(x|x)\log{\bar{P}}_{\sigma }\left(x\right)\right]\;\\ & =\exp\left[-n\left(H\left(P_{x}\right)+D(P_{x}\left|\right|{\bar{P}}_{\sigma })\right)\right] \end{array}$$

holds, a(x) is written as

(A38) $$\begin{array}{cc} a(x) & =\exp\left[-n\left(H\left(P_{x}\right)+v\text{-}\mathrm{ess}.\inf D(P_{x}\left|\right|{\bar{P}}_{\sigma })\right)\right]\;\\ & =\exp\left[-n\left(H\left(P_{x}\right)+D(P_{x}\left|\right|{\bar{P}}_{\sigma (P_{x})})\right)\right]. \end{array}$$

Here, it is important to notice that $D(P||{\bar{P}}_{\sigma })$ is continuous in $\left(P,{\bar{P}}_{\sigma }\right)$ and hence, owing to the assumption, $D(P||{\bar{P}}_{\sigma (Q)})$ is continuous in Q∈P(X). Thus, expanding $D(P||{\bar{P}}_{\sigma (P_{x})})$ in $P_{x}$ around $P_{\theta }$ leads to

(A39) $$\begin{array}{cc} D\left(P||{\bar{P}}_{\sigma (P_{x})}\right) & =D(P||{\bar{P}}_{\sigma (P_{\theta })})+\delta_{\theta }\left(\nu \right)\;\left(x\in T_{\theta ,\nu }^{n}\right). \end{array}$$

with some $\delta_{\theta }\left(\nu \right)$ such that $\delta_{\theta }\left(\nu \right)\to 0$ as ν→0, because $\sum_{x\in X}\left|P_{\theta }\left(x\right)-P_{x}\left(x\right)\right|\leq \nu$ for $x\in T_{\theta ,\nu }^{n}$.

Then, with $P_{x}$ instead of *P* in Equation (A39) and in view of Equation (A36) for each $x\in T_{\theta ,\nu }^{n}$ we have the upper bound:

(A40) $$\begin{array}{cc} P_{{\bar{X}}^{n}}\left(x\right) & \leq a(x)\;\\ & =\exp\left[-n\left(H\left(P_{x}\right)+D(P_{x}||{\bar{P}}_{\sigma (P_{x})})\right)\right]\;\\ & =\exp\left[-n\left(H\left(P_{x}\right)+D(P_{x}\left|\right|{\bar{P}}_{\sigma (P_{\theta })})-\delta_{\theta }\left(\nu \right)\right)\right]\;\\ & ={\bar{P}}_{\sigma (P_{\theta })}^{n}\left(x\right)\exp\left[n\delta_{\theta }\left(\nu \right)\right], \end{array}$$

from which it follows that for each $x\in T_{\theta ,\nu }^{n}$

(A41) $$\begin{array}{cc} \frac{1}{n}\log\frac{1}{P_{{\bar{X}}^{n}}\left(x\right)} & \geq \frac{1}{n}\log\frac{1}{{\bar{P}}_{\sigma (P_{\theta })}^{n}\left(x\right)}-\delta_{\theta }\left(\nu \right). \end{array}$$

Therefore, the proof of Equation (45) has been completed.

(2) Proof of Equation (46):

To prove Equation (46), we show the lower bound of $P_{{\bar{X}}^{n}}\left(x\right)$. For any P∈P(X) and any small constant τ>0, set

(A42) $$\begin{array}{c} S_{\tau }\left(P\right):=\left\{\sigma \in \Sigma \left|D(P||{\bar{P}}_{\sigma })<D(P||{\bar{P}}_{\sigma (P)})+\tau \right.\right\}, \end{array}$$

then, by the definition of *v*-ess.inf,

(A43) $$c_{\tau }\left(P\right):=\int_{S_{\tau }\left(P\right)}dv\left(\sigma \right)>0$$

holds. Our claim is that for any θ∈Θ and sufficiently small τ>0 and with some positive constant $c_{\theta }>0$

(A44) $$\underset{x\in T_{\theta ,\nu }^{n}}{\inf}c_{\tau }\left(P_{x}\right)\geq c_{\theta }.$$

To see this, consider a sequence ${\left\{\tau_{i}\right\}}_{i=1}^{\infty }$ such that $0<\tau_{1}<\tau_{2}<\cdots \to \tau$. Then, there exists a positive integer *m* such that $c_{\tau_{m}}\left(P_{\theta }\right)>0$. Otherwise, the continuity of probability measure implies that

(A45) $$0=\lim_{i\to \infty }c_{\tau_{i}}\left(P_{\theta }\right)=c_{\tau }\left(P_{\theta }\right)>0,$$

which is a contradiction. On the other hand, in view of Equation (A42), $\sigma \in S_{\tau_{m}}\left(P_{\theta }\right)$ is equivalent to

(A46) $$\begin{array}{c} D(P_{\theta }\left|\right|{\bar{P}}_{\sigma })<D(P_{\theta }\left|\right|{\bar{P}}_{\sigma (P_{\theta })})+\tau_{m}, \end{array}$$

(A47) $$\begin{array}{c} D(P_{x}\left|\right|{\bar{P}}_{\sigma })<D(P_{x}\left|\right|{\bar{P}}_{\sigma (P_{x})})+\tau_{m}+\gamma \left(\nu \right)\;\left(\forall x\in T_{\theta ,\nu }^{n}\right), \end{array}$$

where Equation (A47) follows from Equation (A46) by expanding $P_{\theta }$ around $P_{x}$ with some γ(ν)>0 such that γ(ν)→0 as ν→0. Therefore, all $\sigma \in S_{\tau_{m}}\left(P_{\theta }\right)$ satisfy Equation (A47). Now we can take ν>0 so that $\tau_{m}+\gamma \left(\nu \right)<\tau$ to have

(A48) $$\begin{array}{c} D(P_{x}\left|\right|{\bar{P}}_{\sigma })<D(P_{x}\left|\right|{\bar{P}}_{\sigma (P_{x})})+\tau \;\left(\forall x\in T_{\theta ,\nu }^{n}\right). \end{array}$$

Therefore, $S_{\tau_{m}}\left(P_{\theta }\right)\subset S_{\tau }\left(P_{x}\right)$. Hence, we have

(A49) $$0<c_{\tau_{m}}\left(P_{\theta }\right)\leq c_{\tau }\left(P_{x}\right)\;\left(\forall x\in T_{\theta ,\nu }^{n}\right).$$

This is nothing but Equation (A44).

Thus, again for $\forall x\in T_{\theta ,\nu }^{n}$, we have the lower bound

(A50) $$\begin{array}{cc} P_{{\bar{X}}^{n}}\left(x\right) & =\int_{\Sigma }{\bar{P}}_{\sigma }^{n}\left(x\right)dv\left(\sigma \right)\;\\ & \geq \int_{S_{\tau }\left(P_{x}\right)}{\bar{P}}_{\sigma }^{n}\left(x\right)dv\left(\sigma \right)\;\\ & =\int_{S_{\tau }\left(P_{x}\right)}\exp\left[-n\left(H\left(P_{x}\right)+D(P_{x}\left|\right|{\bar{P}}_{\sigma })\right)\right]dv\left(\sigma \right)\;\\ & \geq \int_{S_{\tau }\left(P_{x}\right)}\exp\left[-n\left(H\left(P_{x}\right)+D(P_{x}\left|\right|{\bar{P}}_{\sigma (P_{x})})+\tau \right)\right]dv\left(\sigma \right)\;\\ & =c_{\tau }\left(P_{x}\right)\exp\left[-n\left(H\left(P_{x}\right)+D(P_{x}\left|\right|{\bar{P}}_{\sigma (P_{\theta })})+\tau -\delta_{\theta }\left(\nu \right)\right)\right]\;\\ & \geq c_{\tau_{m}}\left(P_{\theta }\right){\bar{P}}_{\sigma (P_{\theta })}^{n}\left(x\right)\exp\left[n\left(\delta_{\theta }\left(\nu \right)-\tau \right)\right], \end{array}$$

where in the second last equality and in the last inequality we have used the continuity of $D\left(P_{x}\left|\right|{\bar{P}}_{\sigma (P_{x})}\right)$ in $P_{x}$ around $P_{\theta }$ and Equation (A49), respectively. From Equation (A50), we obtain

(A51) $$\begin{array}{cc} \frac{1}{n}\log\frac{1}{P_{{\bar{X}}^{n}}\left(x\right)} & \leq \frac{1}{n}\log\frac{1}{{\bar{P}}_{\sigma (P_{\theta })}^{n}\left(x\right)}+\frac{1}{n}\log\frac{1}{c_{\tau_{m}}\left(P_{\theta }\right)}+\left(\tau -\delta_{\theta }\left(\nu \right)\right), \end{array}$$

for each $x\in T_{\theta ,\nu }^{n}$, which completes the proof of Equation (46). □

## Appendix F. Proof of Theorem 6

First, we prove the inequality:

(A52) $$\begin{array}{c} B_{0}\left(X|\right|\bar{X})\geq \min_{1\leq i\leq K,1\leq j\leq L}B_{0}(X_{i}\left|\right|{\bar{X}}_{j}). \end{array}$$

To do so, we arbitrarily fix $R_{ij}$ for 1≤∀i≤K,1≤∀j≤L so that

(A53) $$R_{ij}<B_{0}(X_{i}\left|\right|{\bar{X}}_{j}).$$

Then, by the definition of $B_{0}(X_{i}\left|\right|{\bar{X}}_{j})$, there exists an acceptance region $A_{n}^{\left(i,j\right)}$ satisfying

(A54) $$\begin{array}{cc} \lim_{n\to \infty }\mu_{n}^{\left(i,j\right)} & =0, \end{array}$$

(A55) $$\begin{array}{cc} \underset{n\to \infty }{lim inf}\frac{1}{n}\log\frac{1}{\lambda_{n}^{\left(i,j\right)}} & \geq R_{ij}, \end{array}$$

where $\mu_{n}^{\left(i,j\right)}$ and $\lambda_{n}^{\left(i,j\right)}$ are defined respectively as

(A56) $$\begin{array}{c} \mu_{n}^{\left(i,j\right)}:=\mathrm{Pr}\left\{X_{i}^{n}\notin A_{n}^{\left(i,j\right)}\right\},\;\lambda_{n}^{\left(i,j\right)}:=\mathrm{Pr}\left\{{\bar{X}}_{j}^{n}\in A_{n}^{\left(i,j\right)}\right\}. \end{array}$$

By using these regions, we define the acceptance region $A_{n}$ as

(A57) $$A_{n}:=\overset{K}{\underset{i=1}{⋃}}\left(\overset{L}{\underset{j=1}{⋂}}A_{n}^{\left(i,j\right)}\right).$$

Then, we have

(A58) $$\begin{array}{cc} \mu_{n}=\mathrm{Pr}\left\{X^{n}\notin A_{n}\right\} & =\sum_{i=1}^{K}\alpha_{i}\mathrm{Pr}\left\{X_{i}^{n}\notin \left(\overset{K}{\underset{i^{\prime }=1}{⋃}}\overset{L}{\underset{j=1}{⋂}}A_{n}^{\left(i^{\prime },j\right)}\right)\right\}\\ & \leq \sum_{i=1}^{K}\alpha_{i}\mathrm{Pr}\left\{X_{i}^{n}\notin \left(\overset{L}{\underset{j=1}{⋂}}A_{n}^{\left(i,j\right)}\right)\right\}\\ & \leq \sum_{i=1}^{K}\sum_{j=1}^{L}\alpha_{i}\mathrm{Pr}\left\{X_{i}^{n}\notin \left(A_{n}^{\left(i,j\right)}\right)\right\}\\ & =\sum_{i=1}^{K}\sum_{j=1}^{L}\alpha_{i}\mu_{n}^{\left(i,j\right)}, \end{array}$$

from which, together with Equation (A54), we obtain

(A59) $$\lim_{n\to \infty }\mu_{n}=0.$$

Similarly, we have

(A60) $$\begin{array}{cc} \lambda_{n}=\mathrm{Pr}\left\{{\bar{X}}^{n}\in A_{n}\right\} & \leq \sum_{j=1}^{L}\sum_{i=1}^{K}\beta_{j}\lambda_{n}^{\left(i,j\right)}, \end{array}$$

from which, together with Equation (A55), we obtain for any small γ>0

(A61) $$\begin{array}{cc} \underset{n\to \infty }{lim inf}\frac{1}{n}\log\frac{1}{\lambda_{n}} & \geq \min_{1\leq i\leq K,1\leq j\leq L}R_{ij}-\gamma . \end{array}$$

Since $R_{ij}$ are arbitrary as far as Equation (A53) is satisfied, we have Equation (A52).

Next, we prove the inequality:

(A62) $$\begin{array}{c} B_{0}\left(X|\right|\bar{X})\leq \min_{1\leq i\leq K,1\leq i\leq L}B_{0}(X_{i}\left|\right|{\bar{X}}_{j}). \end{array}$$

To do so, let *R* be 0-achievable, then there exists an acceptance region $A_{n}$ satisfying

(A63) $$\begin{array}{cc} \lim_{n\to \infty }\mu_{n} & =0, \end{array}$$

(A64) $$\begin{array}{cc} \underset{n\to \infty }{lim inf}\frac{1}{n}\log\frac{1}{\lambda_{n}} & \geq R. \end{array}$$

We fix such an $A_{n}$ and consider the hypothesis testing with null hypothesis $X_{i}$ and alternative hypothesis ${\bar{X}}_{j}$ for arbitrarily fixed *i* and *j*. Then, probabilities of type I error and type II error are given by

(A65) $$\begin{array}{cc} \mu_{n}^{\left(i,j\right)} & =\mathrm{Pr}\left\{X_{i}^{n}\notin A_{n}\right\}, \end{array}$$

(A66) $$\begin{array}{cc} \lambda_{n}^{\left(i,j\right)} & =\mathrm{Pr}\left\{{\bar{X}}_{j}^{n}\in A_{n}\right\}. \end{array}$$

Since

(A67) $$\begin{array}{cc} \mu_{n} & =\sum_{i=1}^{K}\alpha_{i}\mathrm{Pr}\left\{X_{i}^{n}\notin A_{n}\right\}\\ & =\sum_{i=1}^{K}\alpha_{i}\mu_{n}^{\left(i,j\right)}, \end{array}$$

we have

(A68) $$\begin{array}{c} \mu_{n}^{\left(i,j\right)}\leq \frac{\mu_{n}}{\alpha_{i}}. \end{array}$$

From this inequality and Equation (A63) we obtain

(A69) $$\begin{array}{c} \lim_{n\to \infty }\mu_{n}^{\left(i,j\right)}=0. \end{array}$$

Similar to the derivation of Equation (A68), we have

(A70) $$\begin{array}{c} \lambda_{n}^{\left(i,j\right)}\leq \frac{\lambda_{n}}{\beta_{j}}. \end{array}$$

Hence, from Equation (A64) we obtain

(A71) $$\begin{array}{cc} R & \leq \underset{n\to \infty }{lim inf}\frac{1}{n}\log\frac{1}{\lambda_{n}}\\ & \leq \underset{n\to \infty }{lim inf}\frac{1}{n}\log\frac{1}{\lambda_{n}^{\left(i,j\right)}}+\underset{n\to \infty }{lim sup}\frac{1}{n}\log\frac{1}{\beta_{j}}\\ & =\underset{n\to \infty }{lim inf}\frac{1}{n}\log\frac{1}{\lambda_{n}^{\left(i,j\right)}}. \end{array}$$

From Equations (A69) and (A71), it follows that *R* is 0-achievable for the hypothesis testing with $X_{i}$ against ${\bar{X}}_{j}$. Noting that i,j are arbitrary with 1≤i≤K and 1≤j≤L, we obtain

(A72) $$\begin{array}{c} R\leq \min_{1\leq i\leq K,1\leq i\leq L}B_{0}(X_{i}\left|\right|{\bar{X}}_{j}). \end{array}$$

This means that Equation (A62) holds, completing the proof of Theorem 6. □

## Appendix G. Proof of Theorem 8

It suffices to show two inequalities:

(A73) $$\begin{array}{cc} B({\left\{X_{i}\right\}}_{i=1}^{K}||{\left\{{\bar{X}}_{j}\right\}}_{j=1}^{L}) & \leq B({\left\{\alpha_{i},X_{i}\right\}}_{i=1}^{K}||{\left\{\beta_{j},{\bar{X}}_{j}\right\}}_{j=1}^{L}), \end{array}$$

(A74) $$\begin{array}{cc} B({\left\{X_{i}\right\}}_{i=1}^{K}||{\left\{{\bar{X}}_{j}\right\}}_{j=1}^{L}) & \geq B({\left\{\alpha_{i},X_{i}\right\}}_{i=1}^{K}||{\left\{\beta_{j},{\bar{X}}_{j}\right\}}_{j=1}^{L}). \end{array}$$

- *Proof of Equation (A73)*:

Suppose that *R* is 0-achievable for the compound hypothesis testing, that is, there exists an acceptance region $A_{n}$ such that

(A75) $$\begin{array}{cc} \lim_{n\to \infty }\mu_{n}^{\left(i\right)} & =0\;(i=1,2,\cdots ,K), \end{array}$$

(A76) $$\begin{array}{cc} \underset{n\to \infty }{lim inf}\frac{1}{n}\log\frac{1}{\lambda_{n}^{\left(j\right)}} & \geq R\;(j=1,2,\cdots ,L). \end{array}$$

Then, the type I error probability $\mu_{n}$ for the hypothesis testing with mixed general sources is evaluated as follows. By the definition of $\mu_{n}$ and Equation (72), we have

(A77) $$\begin{array}{cc} \mu_{n} & =\mathrm{Pr}\left\{X^{n}\notin A_{n}\right\}\\ & =\sum_{i=1}^{K}\alpha_{i}\mathrm{Pr}\left\{X_{i}^{n}\notin A_{n}\right\}\\ & =\sum_{i=1}^{K}\alpha_{i}\mu_{n}^{\left(i\right)}, \end{array}$$

from which, together with Equation (A75), we obtain

(A78) $$\lim_{n\to \infty }\mu_{n}=0.$$

Similarly, we have

(A79) $$\begin{array}{cc} \lambda_{n} & =\mathrm{Pr}\left\{{\bar{X}}^{n}\in A_{n}\right\}\\ & =\sum_{j=1}^{L}\beta_{j}\mathrm{Pr}\left\{{\bar{X}}_{j}^{n}\in A_{n}\right\}\\ & =\sum_{j=1}^{L}\beta_{j}\lambda_{n}^{\left(j\right)}. \end{array}$$

On the other hand, Equation (A76) implies

(A80) $$\lambda_{n}^{\left(j\right)}\leq e^{-n(R-\gamma )}\;\left(n\geq n_{0}\right),$$

holds for any γ>0 and all j=1,2,⋯,L. Substituting this inequality into Equation (A79) yields

(A81) $$\begin{array}{cc} \underset{n\to \infty }{lim inf}\frac{1}{n}\log\frac{1}{\lambda_{n}} & \geq R-\gamma . \end{array}$$

Since γ>0 is arbitrary, from Equations (A78) and (A81) we conclude that Equation (A73) holds.

- *Proof of Equation (A74)*:

Suppose that *R* is 0-achievable for the mixed hypothesis testing, that is, there exists an acceptance region $A_{n}$ such that

(A82) $$\begin{array}{cc} \lim_{n\to \infty }\mu_{n} & =0, \end{array}$$

(A83) $$\begin{array}{cc} \underset{n\to \infty }{lim inf}\frac{1}{n}\log\frac{1}{\lambda_{n}} & \geq R. \end{array}$$

We fix such an $A_{n}$ and set

(A84) $$\begin{array}{cc} \mu_{n}^{\left(i\right)} & =\mathrm{Pr}\left\{X_{i}^{n}\notin A_{n}\right\}, \end{array}$$

(A85) $$\begin{array}{cc} \lambda_{n}^{\left(j\right)} & =\mathrm{Pr}\left\{{\bar{X}}_{j}^{n}\in A_{n}\right\}. \end{array}$$

Then, from Equation (72) we have

(A86) $$\begin{array}{cc} \mu_{n} & =\sum_{i=1}^{K}\alpha_{i}\mathrm{Pr}\left\{X_{i}^{n}\notin A_{n}\right\}\\ & =\sum_{i=1}^{K}\alpha_{i}\mu_{n}^{\left(i\right)}, \end{array}$$

from which, it follows that

(A87) $$\mu_{n}^{\left(i\right)}\leq \frac{\mu_{n}}{\alpha_{i}}$$

for all i=1,2,⋯,K. From this inequality and Equation (A82), we obtain

(A88) $$\begin{array}{cc} \lim_{n\to \infty }\mu_{n}^{\left(i\right)} & =0, \end{array}$$

for all i=1,2,⋯,K. Similarly,

(A89) $$\begin{array}{cc} \lambda_{n} & =\sum_{j=1}^{L}\beta_{j}\mathrm{Pr}\left\{{\bar{X}}_{j}^{n}\in A_{n}\right\}\\ & =\sum_{j=1}^{L}\beta_{j}\lambda_{n}^{\left(j\right)}, \end{array}$$

so that we have for j=1,2,⋯,L,

(A90) $$\lambda_{n}^{\left(j\right)}\leq \frac{\lambda_{n}}{\beta_{j}},$$

which means that

(A91) $$\begin{array}{cc} \frac{1}{n}\log\frac{1}{\lambda_{n}^{\left(j\right)}} & \geq \frac{1}{n}\log\frac{\beta_{j}}{\lambda_{n}}\;\\ & =\frac{1}{n}\log\frac{1}{\lambda_{n}}-\frac{1}{n}\log\frac{1}{\beta_{j}}. \end{array}$$

Noting that $\beta_{j}\;\left(j=1,2,\cdots ,L\right)$ are constants, from Equation (A83) we obtain

(A92) $$\begin{array}{cc} \underset{n\to \infty }{lim inf}\frac{1}{n}\log\frac{1}{\lambda_{n}^{\left(j\right)}} & \geq R, \end{array}$$

for all j=1,2,⋯,L. From Equations (A88) and (A92), we conclude that Equation (A74) holds. □

## References

[1] Han T.S. (2003). Information-Spectrum Methods in Information Theory.

[2] Dembo A., Zeitouni O. (1993). Large Deviations Techniques and Applications.

[3] Chen P.N. (1996). General formulas for the Neyman-Pearson type-II error exponent subject to fixed and exponential type-I error bound. IEEE Trans. Inf. Theory.

[4] Strassen V. Asymptotische Abshätzungen in Shannon’s Informations Theorie. Proceedings of the Transactions of the 3rd Prague Conference on Information Theory.

[5] Hayashi M. (2009). Information Spectrum Approach to Second-Order Coding Rate in Channel Coding. IEEE Trans. Inf. Theory.

[6] Polyanskiy Y., Poor H., Verdú S. (2010). Channel Coding Rate in the Finite Blocklength Regime. IEEE Trans. Inf. Theory.

[7] Nomura R., Han T.S. (2013). Second-Order Resolvability, Intrinsic Randomness, and Fixed-Length Source Coding for Mixed Sources: Information Spectrum Approach. IEEE Trans. Inf. Theory.

[8] Tan V., Kosut O. (2014). On the Dispersion of Three Network Information Theory Problems. IEEE Trans. Inf. Theory.

[9] Watanabe S. (2017). Second-Order Region for Gray–Wyner Network. IEEE Trans. Inf. Theory.

[10] Polyanskiy Y., Poor H.V., Verdú S. (2011). Dispersion of the Gilbert-Elliott Channel. IEEE Trans. Inf. Theory.

[11] Nomura R., Han T.S. (2014). Second-Order Slepian-Wolf Coding Theorems for Non-Mixed and Mixed Sources. IEEE Trans. Inf. Theory.

[12] Yagi H., Han T.S., Nomura R. (2016). First- and Second-Order Coding Theorems for Mixed Memoryless Channels with General Mixture. IEEE Trans. Inf. Theory.

